# Engineering conditional peptide self-assembly using AlphaFold

**DOI:** 10.1016/j.xcrp.2026.103551

**Published:** 2026-09-10

**Authors:** Helen Futty, Sofia Chinelli, Ravi R. Sonani, Tomás S. Plivelic, Annela Seddon, Edward H. Egelman, Andrew R. Thomson, Dave J. Adams

**Affiliations:** 1 School of Chemistry, University of Glasgow, Glasgow G12 8QQ, UK; 2 Department of Chemistry “Giacomo Ciamician”, University of Bologna, Via Piero Gobetti 85, 40129 Bologna, Italy; 3 Department of Biochemistry and Molecular Genetics, University of Virginia, Charlottesville, VA 22903, USA; 4 MAX IV Laboratory, Lund University, Fotongatan 2, 224 84 Lund, Sweden; 5 HH Wills Physics Laboratory, University of Bristol, Tyndall Avenue, Bristol BS8 1TL, UK; 6 Lead contact

## Abstract

Programming self-assembly from peptide sequence remains difficult because existing design rules are only weakly predictive of emergent supramolecular behavior. Here, we show that AlphaFold Multimer can be used to engineer conditional peptide self-assembly by simultaneously selecting for co-assembly and against assembly of the individual components. Using model confidence metrics as proxies for assembly propensity, we designed peptide pairs that largely remain unstructured in isolation but form defined tape-like nanostructures upon mixing. Experimental measurements including circular dichroism, small-angle X-ray scattering, and cryo-electron microscopy validate the predicted behavior across multiple systems. These results establish artificial intelligence-guided design as a practical route to programmable supramolecular peptide materials.

## In brief

Futty et al. demonstrate a method of using AlphaFold as a design tool for targeted assembly and non-assembly of short peptides. Through an AlphaFold-guided rational design process, several self-assembling peptide systems are designed, and AlphaFold identifies where subtle differences in amino acid sequences lead to significant changes in assembly behavior.

## Graphical abstract

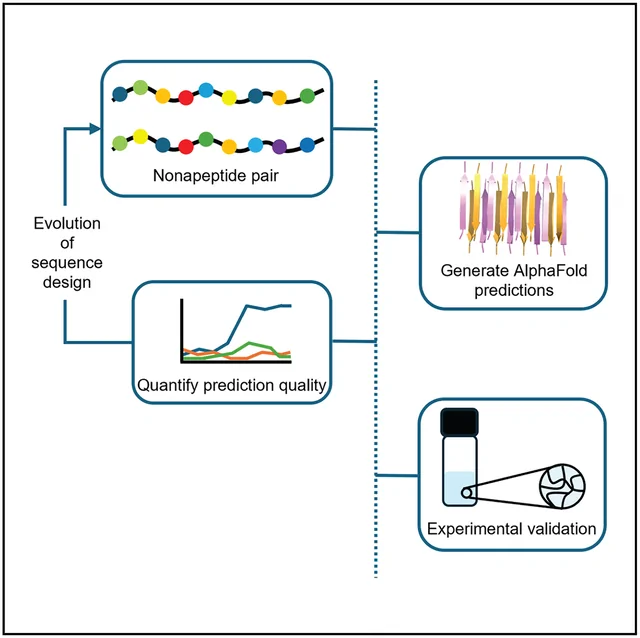

## INTRODUCTION

The self-assembly of short peptides has been shown to be an effective method of preparing a range of soft materials with uses in areas including cell culturing, drug delivery, tissue engineering, optoelectronics, and sensing.^1–6^ Many different oligopeptides have been described,^7–10^ including classes such as functionalized amino acids and dipeptides,^11–17^ charge complementary peptides,^18–20^ peptide amphiphiles,^21,22^ collagen mimics,^23–25^ and α-helical peptides.^26^ A number of these materials are now commercialized, showing the great potential here. In all cases, the peptides self-assemble by non-covalent interactions into primary fibrillar nanostructures, which then build up to form the materials. This self-assembly is pre-programmed into the system via the sequence of amino acids within the peptide.^27,28^

Underpinning all of these are design rules that have been determined over the last 40 or so years.^29^ Indeed, many of these rules have not been amended or updated for many years yet have proven to be sufficiently robust to allow an understanding of many of the current systems. These design principles include an understanding that specific amino acids favor the formation of specific secondary structures, that the pattern of hydrophobic and hydrophilic amino acids can be used to drive the formation of α-helices or β-sheets for example,^30–33^ and that further interactions such as charge bridges can be used to control the self-assembly.

However, these design rules are in many cases more used to post-rationalize the outcome, with many sequences used being analogues of other examples. Beyond these relatively simple design rules, computational approaches are showing some success in terms of designing specific structures.^34–38^ As things stand, the known design rules are rather coarse tools that allow the selection of specific secondary structures, but which are poorly predictive of the degree of self-assembly. New approaches are therefore needed in order to develop smarter peptide materials. Herein, we test the use of AlphaFold in the development of a two-peptide material system that exhibits conditional self-assembly only when both components are present. The key motivation to examine such systems is that many peptide materials are currently based on single-component systems, where the molecular sequence must simultaneously encode solubility, self-assembly, material properties, and function. This inevitably limits the accessible design space. In contrast, conditional co-assembly separates these requirements across two independently soluble building blocks, allowing assembly to occur only when both components are present. This introduces an additional level of control over when and where assembly occurs while greatly expanding the number of accessible combinations of molecular interactions.

Artificial intelligence-based tools have revolutionized protein science. AlphaFold^39^ has been shown to be highly effective in terms of predicting protein structure, and AlphaFold Multimer^40,41^ (AF-M) can accurately predict protein:protein and protein:peptide complexes. There are also a growing number of examples where AlphaFold has been used to predict the structure of short peptides and their assemblies with varying degrees of success.^42–45^ We hypothesized that we could use this tool to predict *a priori* the co-assembly of two peptides and importantly, the non-assembly of the individual peptides. This would enable a new approach to the design and use of small peptides as building blocks for useful materials. Because an individual prediction can be made in minutes, this approach has the potential to hugely accelerate the design of new peptide materials.

The purpose of AlphaFold Multimer in this work is neither to predict the complete self-assembly landscape nor to calculate thermodynamic stability or kinetic accessibility. Instead, we use the confidence metrics as a practical means of identifying peptide combinations that are likely to adopt well-defined intermolecular arrangements while simultaneously selecting against stable assemblies of the individual peptide components. The experimental validation by circular dichroism (CD), small-angle X-ray scattering (SAXS), and cryo-electron microscopy (cryo-EM) is then used to determine whether these predicted assemblies are realized experimentally.

## RESULTS AND DISCUSSION

Initially, we tested AlphaFold’s capacity to recapitulate the structures of known self-assembling oligopeptide systems. We focused predominately on predicted local distance difference test (pLDDT) scores as a metric for the confidence in each prediction, which is a score out of 100 of the local confidence in the positioning of each amino acid residue. Predicted template modeling (pTM), interface predicted template modeling (ipTM), and predicted aligned error (PAE)^39^ were also examined but were not used in quantitative analysis of prediction quality. High-resolution structural data are rare for these systems. However, AlphaFold reproduces the double-layer beta hairpin structure of MAX-1^46^ with high confidence (average pLDDT >95). Importantly, despite this correct prediction for MAX-1, only 4 of the 10 models produced were of high confidence and only two showed the correct structure (Figure S1). The remainder of the models were low confidence, with pLDDT values in the range of 70–30, and demonstrated various off-target “misfolded” topologies, such as a large β-barrel. Thus, structure prediction of multi-peptide assemblies has a comparatively high failure rate. To account for this, we have taken the approach of generating large numbers of models for each system (5 models for 8 seeds, giving 40 models in total). Predicted structures of other β-sheet-forming systems such as FEFEFKFK and Q-11 (with amino acid sequence QQKFQFQFEQQ) are consistent with reported low-resolution data.

It is important to consider how short peptides differ from the globular proteins in AlphaFold’s training set, as this has a large impact on prediction quality. Limited sampling results in a wide range of confidence values across predicted structures, so to eliminate any uncertainty, we chose to guide the design process using a threshold of pLDDT >90 for “good”-quality predictions. This threshold was selected empirically during method development because it provided a clear separation between systems that consistently generated well-defined structures and those that did not.

AlphaFold Multimer predicts static structural models and does not explicitly account for solvent, dynamics, competing assembly pathways, or kinetic trapping. We emphasize that experimental validation remains an essential component of the workflow and that the computational predictions are intended to reduce the sequence space requiring experimental investigation rather than replace experiment. As such, we do not use pLDDT as a direct measure of thermodynamic stability, binding affinity, or assembly free energy. Rather, we use it as a practical screening metric to identify peptide combinations that consistently generate well-defined intermolecular arrangements while simultaneously rejecting systems that readily assemble as individual components.

### An AlphaFold-guided rational design process

Using these simple measures, we set out to use AF-M to guide the design of a two-component system, where each individual peptide does not form extended self-assembled structures, but interactions between the two components would result in the formation of fibers. We targeted a two-component tape-like fiber consisting of anti-parallel aligned peptide strands. High-confidence models were taken to be a prediction of topology, and the inability to generate a high-confidence model was taken to be a prediction of a low capacity to self-assemble. As such, in the peptide design process shown in Figure 1, systems with average pLDDT <90 were discarded. Systems which displayed predictions with pLDDT >90 were also required to have several models with this property (Figure S2). Peptide pairs that displayed high-confidence predicted structures were also studied across a range of oligomeric states, and a system was deemed reliable only if the structure was consistent and high-confidence for several different numbers of strands. To carry out the oligomer scans, we ran predictions on systems containing 4 to 16 strands. Though theoretically this scan could be carried out to much larger oligostates, we chose to end the scan at 16 strands, as at this point the predicted structures of the β-sheet assemblies are consistently high-confidence, and the computational cost is not too high. Here, we are using the oligomer scan to assess the consistency of the predicted structure rather than make any findings about large-scale supramolecular behavior, and we do not base any dimensions of nanostructures on any oligostate prediction. Based on literature precedent,^29,47–49^ peptides were designed with alternating hydrophilic and hydrophobic groups to promote β-strand formation. To target peptides that would not self-assemble effectively individually, we focused on hydrophobic cores of marginal stability. Our overall design principle was to build sequences in which a moderately hydrophobic alanine-rich core would be supported by other interactions only in the binary combination of the two peptides. Accordingly, we explored patterns of charged glutamate and lysine residues, as well as β-sheet-favoring threonine at the solvent-facing positions. For the hydrophobic positions, we explored combinations of alanine and valine residues. Potential sequences were modeled using AF-M, and the number of high-confidence models were enumerated for both the individual and mixed peptides (Figure 1; for peptide nomenclature, see Table S1). As exemplified by the predictions of the all-valine core peptide system (peptides **1a** and **1b**), too much hydrophobicity leads to high confidence in the predictions of the individual peptide KVEVKVEVK (**1b**) (Figure S3), which diminishes when this is mutated to an alternating alanine and valine core (peptides **2a** and **2b**) (Figure S4). Incorporating threonine residues into the sequences increases confidence in the two-component fiber (peptides **3a** and **3b**) (Figure S5). We believe that the high-confidence barrel-like structures observed in the predictions of EVTAKVKAT (**3b**) as a 16-mer are an artifact that can be treated as improbable due to the large hydrophobic cavity in the core of the structure, a feature not exhibited by other predictions in this system (Figure S5). Aromatic residues have been shown to stabilize strand-strand interactions.^50–52^ We therefore included a tryptophan residue in each peptide sequence such that a contiguous strip of aromatic interactions could be formed only in the binary combination. These considerations led us to the system EVWAKVKAT (peptide **4a**) and TATVEAWVK (peptide **4b**), for which seven of 40 models had a pLDDT value above 90 for the binary combination (Figures S2 and S6). Through this design process, we have used AlphaFold to identify which amino acids play key roles in the confidence metrics for the predicted structures. These findings could be experimentally validated through a mutational analysis study that would be an extensive follow-up to the findings presented here.

The predictions of the individual peptides for **4a** and **4b** did not display any consistent structure for models of 4–16 strands and typically had pLDDT values <60. A plot of the number of strands against average pLDDT value of the top ranked model for each prediction shows a large and consistent gap between the two-peptide and one-peptide systems (Figure 1D). Taken together, the low-confidence models and variable predictions suggest that the individual **4a** and **4b** peptides do not have a well-defined structure. In contrast, the two-component system is predicted with high confidence to form the target tape-like structure, and this structure is consistently predicted when three or more of each strand are present (Figure 1D). A 100 ns molecular dynamics run of the **4a** + **4b** model showed no change in topology or unfolding, supporting the prediction that this is a stable structure (Figure S9).

### Experimental validation of AlphaFold predictions

Experimentally, all peptides here were prepared by solid-phase synthesis. Purities were typically >80% and most about 90% (Figures S10–S14). While we cannot completely exclude a minor contribution from these impurities, we believe they are unlikely to influence the principal conclusions for two reasons. First, all peptide systems were prepared and characterized using identical synthetic and purification procedures. Second, the clear and reproducible differences observed between closely related peptide sequences indicate that the assembly behavior is determined by the designed sequence changes rather than low-level impurities.

We focus experimentally on a concentration of 10 mM. This concentration was selected to ensure robust assembly and facilitate characterization by CD, SAXS, and cryo-EM using a common set of experimental conditions. Our objective was to validate the computational workflow rather than determine the critical aggregation concentration or concentration dependence of each peptide system. The computational design strategy itself is independent of concentration. We therefore expect the workflow to remain applicable at lower concentrations, although the experimental observation of assembly will naturally depend on the concentration regime appropriate for the particular peptide system under investigation.

Both **4a** and **4b** as single-component systems resulted in (CD) spectra that showed a lack of structure across a pH range from 2 to 12, and at a concentration of 10 mM, with all solutions being transparent (Figures 2A–2C; Figure S15). Cryo-EM showed an absence of any self-assembled structures, and SAXS data showed that these samples scatter poorly, demonstrating the absence of any extended self-assembled structures (Figures 2D and 2E). In comparison, on mixing a 1:1 ratio of **4a** and **4b**, a turbid solution was formed after overnight stirring at pH 7; at the extremes of pH, transparent solutions were formed as expected given the predicted peptide charges^53^ (Figure S24). We acknowledge that the ionic strength and counter-ion effect can play a major role in peptide assembly. All peptide solutions were prepared using an identical method as outlined in the Methods section, but further work investigating pH-dependent behavior in buffers of constant ionic strength would provide a more in-depth analysis of solution thermodynamics. CD spectra of the mixed solution show clear secondary structure signals; however, tryptophan signals dominate the spectra, making interpretation of secondary structure challenging (Figures 2A–2C; Figures S16 and S18). FTIR confirms β-like structures are present (Figure S23). SAXS data showed that highly scattering solutions were formed at pH 7; the data were best fit to an elliptical cylinder model (Figures 2D; Table S2). In agreement with these fits to the data, the cryo-EM shows the presence of tape-like structures (Figure 2E). The turbidity shown in Figure 2C implies large aggregates. The cryo-EM images consistently show the tape-like nanostructures expected from the SAXS measurements, indicating that these assemblies dominate the nanostructure of the sample. It is likely that the high density of such structures leads to the scattering of light and giving the observed turbidity.

The specificity of this structure formation was investigated by a CD Job plot (Figure 2B), which displayed a strongest CD signal at 208 nm at a 1:1 ratio (Figure S17). This wavelength was selected as the global maximum exhibited at 208 nm is a prominent peak in all CD spectra, where the change in intensity is clear across the range of solutions.

### AlphaFold distinguishes between subtly different amino acid sequences

In addition to this peptide system, several other peptide systems were designed and were predicted to form a similar tape-like structure. The system EVWAKVKAT (**4a**) + TATVEAWVT (**5b**) exhibits low confidence predictions for the individual peptides across a range of number of peptide chains, while the two-component system is predicted to form a double-layered structure similarly to the **4a** + **4b** system (Figure 3A). The two-component system consists of 10 of the 40 models with average pLDDT >90, and the structure is recapitulated with high confidence after 10 peptide strands are used in a prediction (Figures S2 and S7). Experimentally, the mixed solution exhibits similar CD features at pH 7 to **4a** + **4b** indicating similar molecular packing, while the individual peptides are likewise unstructured (Figures 3A and 3B; Figure S19). A Job plot by CD at 230 nm (Figure 3C) of this peptide system shows 1:1 specificity (Figure S20). Cryo-EM images show loose aggregates present in solutions of **4a**, and nanotube structures in solutions of **5b**, despite the unstructured CD signals. CD primarily reports on local molecular organization and secondary structure rather than higher-order supramolecular morphology. Consequently, two systems may exhibit very similar CD spectra despite assembling into different mesoscale structures. These nanostructures present in the cryo-EM images differ from the tape-like structures present in the two-component solution, which is consistent with the AlphaFold predictions and CD signals, indicating that these local packing motifs subsequently organize into distinct supramolecular morphologies. SAXS data show the individual peptide solutions scatter minimally, indicating that the tubular structures picked up by cryo-EM are small, localized pockets of aggregation (Figures 3D and 3E; Table S3). Peptide **5b** was challenging to synthesize and exhibited larger impurity peaks by HP-LC (Figure S12). It is possible that these large nanostructures present in cryo-EM images are due to aggregation of impurities. The two-component solution fits to an elliptical cylinder model, similarly to **4a** + **4b**, which is consistent with the tape-like structures visible in the cryo-EM images (Figures 3D and 3E). A similar system, KVWAKVEAT (**6a**) + TAKVEAWVT (**6b**), was designed to minimize end-on stacking of peptide chains by reducing the charge at the ends of the sequences (Figure 3F). This involved swapping positions 1 and 7 in peptide **4a** and 3 and 9 in peptide **4b**. However, with this change, **6b** is predicted to form fibrous structures with high confidence (Figure 3F). The predictions even out-perform those of the two-component system by pLDDT scores, indicating that the assembly of **6b** strands is competing with the co-assembly of the two peptides. The two-component prediction consists of two models with pLDDT >90 (Figures S2 and S8), while the prediction of **6b** consists of one model with pLDDT >90 (Figure S8). While the CD spectra of both **6b** and **6a** + **6b** solutions show similar line shapes, there is a blue shift in signals observed for **6b** (Figures 3G; Figure S21). This indicates similar, but not identical, molecular packing. Moreover, the band observed at 280–300 nm in **6a** + **6b** spectra is not present in **6b** spectra. As this band arises in the near-UV region, it must be a result of tryptophan excimer formation. The lack of this signal in **6b** spectra is consistent with the predicted anti-parallel structure that places tryptophan residues at opposite ends of the fiber. A Job plot by CD at 235 nm (Figure 3H) of this system shows a preference toward increased concentrations of **6a** (Figure S22). Cryo-EM images show thin fibers present for **6a** (Figure 3I); however, minimal scattering is observed in the SAXS data (Figures 3J; Tables S4 and S5). Flat tape-like fibers are visible in the cryo-EM images of **6b** and **6a** + **6b**, which both have SAXS data fitting to an elliptical cylinder model. The dimensions of the fit for the two-component solution are similar to those observed for the **4a** + **4b** system. Hence, we can use AlphaFold to distinguish between subtle differences in amino acid sequence, which would not be possible by rational design alone.

In conclusion, we have shown that rational design principles, guided by AF-M models, allow the design of two-component peptide materials from first principles. This approach allows the rapid screening of designs ahead of experimental evaluation. Conditional co-assembly can be programmed at the sequence level by targeting non-assembly of individual peptides as a design parameter. While AF-M does not directly predict stability, model confidence metrics can be used as effective proxies of assembly propensity. The method is limited upon the transition from molecular-scale packing to larger supramolecular assemblies, as AlphaFold models do not capture how the secondary structures aggregate together. However, all systems predicted to form a β-sheet structure consistently exhibit tape-like structures in cryo-EM images and SAXS data, reflecting how these types of secondary structures tend to associate together. In principle, the methodology is not restricted to this specific peptide class. AlphaFold Multimer has already demonstrated impressive predictive capability for a range of protein-protein interactions and has shown success for several classes of peptide assemblies. Consequently, provided sufficiently well-defined intermolecular structures exist, we envisage that the workflow could also be applied to other supramolecular systems including coiled coils, collagen-like assemblies, and other peptide architectures. However, we also recognize that systems with significant structural heterogeneity, extensive conformational disorder, or highly dynamic assembly pathways are likely to be considerably more challenging. Nevertheless, we believe that the success rate of sequence exploration afforded by this method will greatly exceed that of rational design alone and will enable the development of peptide materials with targeted condition-specific properties. We envisage this workflow not as a replacement for physical models of peptide self-assembly, but as a practical sequence-screening strategy. By rapidly reducing the accessible sequence space to a manageable number of experimentally testable candidates, AlphaFold Multimer provides an efficient route for identifying promising conditional co-assembling systems, while detailed mechanistic understanding can subsequently be developed using structural, spectroscopic, and computational approaches. The methodology should be applicable to a broader range of peptide lengths, although the optimum design criteria may differ between different peptide classes.

## METHODS

### Materials

L-amino acids, ethyl cyanohydroxyiminoacetate (Oxyma), N,N-diisopropylcarbodiimide (DIC), trifluoroacetic acid (TFA), and triisopropyl silane (TIPS) were purchased from Fluorochem. Fmoc-Lys(Boc)-Wang resin (0.29 mmol g^−1^ loading) was purchased from CEM. Fmoc-Thr(tBu)-Wang resin (0.64 mmol g^−1^ loading) was purchased from Merck. Morpholine (≥ 99%), diethyl ether (≥ 99%), and sodium hydroxide (NaOH) were purchased from Sigma Aldrich. N,N-dimethylformamide (DMF) was purchased from Rathburn. Dichloromethane (DCM) and acetonitrile (≥ 99.8%) (MeCN) were purchased from Thermo Fisher Scientific. Hydrochloric acid (36.5%–38%) (HCl) was purchased from Honeywell Fluka. Deionized H_2_O was used throughout.

### AlphaFold2 predictions

Peptide structures were predicted using ColabFold v.1.5.5 implementing AlphaFold2-Multimer (GitHub—Sokrypton/ColabFold)^39^ or LocalColabFold (GitHub—YoshitakaMo/localcolabfold) run on an Ubuntu Linux workstation equipped with an Nvidia RTX4080 GPU.

For predictions of MAX-1, the following parameters were used: Amber relaxation on 5 models, mmseqs2_uniref_env MSA mode, model type alphafold_multimer_v3, rank by pLDDT, 20 recycles, 2 seeds incrementing from 0000 (5 models per seed), producing 10 models.

Predictions of all other peptide systems were run with the following parameters: Amber relaxation on 5 models, mmseqs2_uniref_env MSA mode, model type alphafold_multimer_v3, rank by pLDDT, 20 recycles, 8 seeds incrementing from 0000 (5 models per seed), producing 40 models. For individual peptides, predictions were run of 4 up to 16 chains to identify the number of strands with the highest confidence prediction. For two-component systems, models were run of peptide A + peptide B in the following concatenations: 2 + 2, 3 + 3, 4 + 4, 5 + 5, 6 + 6, 7 + 7, and 8 + 8.

Each model produced in an AlphaFold2 prediction comes with a “scores” file, which contains the pLDDT and PAE values across the entire predicted structure. Using these values, average pLDDT scores were calculated for each model.

### Molecular visualization

AlphaFold2-predicted structures come as PDB files, which were visualized using the molecular visualization software PyMOL 3.1. To color structures according to their pLDDT values, the command

set_color n0, [0.051, 0.341, 0.827]
set_color n1, [0.416, 0.796, 0.945]
set_color n2, [0.996, 0.851, 0.212]
set_color n3, [0.992, 0.490, 0.302]
color n0, b < 100; color n1, b < 90
color n2, b < 70; color n3, b < 50

was used.

### Peptide synthesis

Peptides were synthesized using a CEM Liberty Blue peptide synthesizer, using DIC/Oxyma activation and 20% morpholine in DMF for Fmoc deprotection. Peptides were synthesized on a 0.1 mmol or 0.5 mmol scale using polystyrene-Wang resins pre-loaded with the C-terminal amino acid in the peptide sequence.

For 0.1 mmol synthesis, the following parameters were selected: synthesis scale: 0.10; is UV; C-terminus: acid; resin type: pre-loaded; resin-swelling: high swelling; resin cycle: 0.10-resin-swelling (HS); final deprotection cycle: 0.1-final deprotection (HS) (UV); couple cycle: 0.10 single ART (HS) (UV).

For 0.5 mmol synthesis, the following parameters were selected: synthesis scale: 0.50; is UV; C-terminus: acid; resin type: pre-loaded; resin-swelling: high swelling; resin cycle: 0.50-resin-swelling (HS); final deprotection cycle: 0.50-final deprotection (HS) (UV); coupling cycle: 0.50 UK single coupling (HS) (UV).

After synthesis, the peptide-loaded resin was transferred to a 10 mL plastic syringe fitted with a filter and syringe tap. With this set-up, the resin was washed with DCM four times to remove DMF. On the 0.1 mmol synthesis scale, cleavage was carried out in the plastic syringe. On the 0.5 mmol synthesis scale, cleavage was carried out in a 100 mL round-bottom flask. Peptides were cleaved from the resin using a TFA/TIPS/H_2_O cleavage cocktail in a 95:2.5:2.5 ratio with a total volume of 5 mL for 0.1 mmol synthesis, and 25 mL for 0.5 mmol synthesis. Where the cleavage was carried out inside the plastic syringe, the mixture was shaken lightly, then the syringe plunger tilted to remove initial CO_2_ produced from Boc deprotection. The resin and cleavage cocktail were left for 2–4 h on a Thermo Scientific Tube Revolver Rotator for the plastic syringe or on a magnetic stirrer plate for the round-bottom flask (stirring for the first 30 min, followed by intermittent stirring over the remaining time to avoid crushing and releasing resin into the mixture). Following this, the cleavage cocktail containing cleaved peptide was filtered from the resin. Peptides were precipitated in a 50 mL falcon tube (or 6 × 50 mL falcon tubes for the 0.5 mmol synthesis) using a 10-fold excess of ice-cold diethyl ether for up to 30 min, followed by 5 min of centrifugation. The diethyl ether was poured off and the pellet left at the bottom of the tube was re-dissolved in 1:1 H_2_O/acetonitrile (MeCN) and freeze-dried.

When high-performance liquid chromatography (HPLC) and mass spectrometry showed the presence of CO_2_ adducts commonly formed when synthesizing tryptophan-containing peptides, additional freeze-dry cycles were required. These were carried out until the CO_2_ adduct peaks disappeared.

### HPLC

Analytical HPLC to verify sufficient purity was performed on either a Shimadzu reverse-phase HPLC system, equipped with Shimadzu LC-20AT pumps, a Shimadzu SIL-20A autosampler, Shimadzu SPD-20A UV-Vis detector monitoring at 214 nm and 280 nm, with a Phenomenex Aeris 5 μm peptide XB-C18 150 × 4.6 mm column and run at a flow rate of 1 mL min^−1^, or a Dionex UltiMate 3000 UHP-LC system, equipped with a Dionex UltiMate 3000 pump, Dionex UltiMate 3000 autosampler, and Ultimate 3000 diode array detector, equipped with a Gemini 5 μm C18 110 Å 150 × 2 mm column and run at a flow rate of 0.3 mL min^−1^. Samples were prepared by dissolving minimal peptide in H_2_O. Gradients were run using a binary solvent system consisting of solvent A (H_2_O + 0.1% TFA) and solvent B (MeCN +0.1% TFA). Analytical HP-LC data are reported as column retention time in minutes (min), along with the gradient used.

### LC-MS

Liquid chromatography-mass spectrometry (LC-MS) analysis to verify mass of product was performed on either an Agilent Infinity Lab liquid chromatography mass spectrometer, using positive mode electrospray ionization (ESI^+^), fitted with a Gemini 5 μm C18 110 Å, 150 × 2 mm LC column at a flow rate of 0.5 mL min^−1^ or an Agilent technologies 1200 series liquid chromatography mass spectrometer using a triple quad mass spectrometer with positive mode electrospray ionization (ESI^+^), fitted with a Restek Rok C18 5 μm 150 × 3.0 mm column at a flow rate of 0.5 mL min^−1^. Samples were prepared by dissolving minimal peptide in H_2_O. Linear gradients were run using a binary solvent system of buffer A (H_2_O + 0.1% formic acid) and buffer B (MeCN +0.1% formic acid) over 20 min. Analytical LC-MS data are reported as counts against mass to charge, along with the gradient used and the expected mass.

### MALDI-TOF

MALDI-TOF spectrometry to verify mass of product was performed on a Shimadzu MALDI-TOF (MALDI8030) spectrometer. Samples were prepared by dissolving minimal peptide in H_2_O, then 0.5 μL was spotted onto a Shimadzu FlexiFocus slide along with 0.5 μL 2,5-dihydroxybenzoic acid matrix dissolved in 1:1 H_2_O and MeCN. The spectrometer was calibrated using 0.5 μL Shimadzu TOFmix with 0.5 μL α-cyano-4-hydroxycinnamic acid matrix.

### Preparation of peptide solutions

Solutions were prepared by weighing the peptide accurately into a vial and making up to the target concentration using an appropriate number of equivalents of 0.1 M NaOH solution to reach the target pH and deionized water. For pH 2, 0 equivalents of NaOH were used. For pH 7, 2.5 equivalents of NaOH were used. For pH 12, four equivalents of NaOH were used. The solution was left to stir overnight (between 16 and 24 h) using a 13 × 3 mm octagonal stirrer bar, and pH was adjusted to the exact target pH ± 0.1 using 1 M NaOH and 1 M HCl the following day.

### Standard preparation of two-component solutions

For two-component solutions of peptides in a 1:1 ratio, equal volumes of each peptide solution (prepared as above) were transferred to a vial with a 13 × 3 mm octagonal stirrer bar, diluted with H_2_O if required, and left to stir overnight.

For solutions of the peptides in ratios differing from 1:1, calculated volumes of peptide solution and H_2_O (if dilution was required) were pipetted into a vial with a 13 × 3 mm octagonal stirrer bar and left to stir overnight.

### Preparation of adjusted two-component peptide solutions

Peptides were weighed accurately into a vial and made up to the target concentration using deionized H_2_O. The pH of the dissolved solution was between 2 and 4. These were left to stir overnight using a 13 × 3 mm octagonal stirrer bar, then adjusted up to pH 7 using 1 M NaOH the following day. The volume of NaOH added was sufficiently small that the effect on the concentration was negligible. An equal volume of each pH 7 peptide solution was transferred to a vial with a 13 × 3 mm octagonal stirrer bar then left to stir overnight.

### Preparation of “mix then adjust” two-component peptide solutions

Peptides were weighed accurately into a vial and made up to the target concentration using deionized H_2_O. These were left to stir overnight with a 13 × 3 mm octagonal stirrer bar, then equal volumes of each peptide solution were transferred to a vial. This was left to stir for at least 5 h using a 13 × 3 mm octagonal stirrer bar. The resulting two-component peptide solution had a pH between 2 and 4. This solution was adjusted up to pH 7 using 1 M NaOH the following day, then left to stir overnight.

### Preparation of two-component peptide solutions by slow addition

Solutions of individual peptides were prepared as described above, then 0.75 mL of peptide A pH 7 10 mM was transferred to a Sterilin vial; 0.15 mL of peptide B pH 7 10 mM was pipetted into the vial in 5 increments, with 1.5 h stirring between additions. After the fifth addition, the solution was left to stir overnight.

### Circular dichroism measurements

CD measurements were performed on an Applied Photophysics Chirascan VX circular dichroism spectropolarimeter, equipped with a Quantum Northwest TC temperature controller and a CW-3000 industrial chiller.

CD spectra were recorded at 20°C on a wavelength range of 180–400 nm with scanning step size of 1 nm and scanning rate of 0.25 s unless stated otherwise. Peptide solutions were prepared as stated above at the desired concentration or diluted from a stock solution and measurements were taken using a demountable quartz 0.01 mm cuvette unless stated otherwise.

All CD data are presented as ellipticity and recorded in millidegrees (mdeg). Absorbance and high-tension (HT) spectra were recorded with CD spectra. All spectra were recorded in duplicate, then averaged and background-corrected.

For a job plot by CD, spectra were measured of two-component peptide solutions at a total concentration of 10 mM with individual peptide concentrations of 0 mM + 10 mM, 1 mM + 9 mM, 2 mM + 8 mM, 3 mM + 7 mM, 4 mM + 6 mM, 5 mM + 5 mM, 6 mM + 4 mM, 7 mM + 3 mM, 8 mM + 2 mM, 9 mM + 1 mM, and 10 mM + 0 mM, respectively, for peptide A + peptide B. Scans were run in duplicate, then averaged. The CD intensity was measured at an appropriate point in the spectra and plotted against peptide A + peptide B ratio.

### Small-angle X-ray scattering measurements

SAXS measurements of peptides **4a** and **4b** were performed at the CoSAXS beamline at the 3 GeV ring of the MAX IV Laboratory, Lund, Sweden. The X-ray beam had energy of 12.4 keV, and the Eiger 2 × 4M detector was placed 1.737 m from the sample. Solutions prepared as described above were transferred to 80 × 1.5 mm (wall thickness 0.01 mm) borosilicate capillaries using a 1 mL syringe and 21 G needle and sealed using UV cured glue. A capillary with H_2_O was also prepared and measured as a background. Each capillary was exposed to 0.5 s radiation. PyFAI^54^ was used for sample-detector distance calibration. The raw data were processed using the Dawn Science 2.27^55,56^ software, involving calibration, masking, background subtraction, and a full azimuthal integration to reduce the 2D images to 1D intensity vs. Q plots.

Measurements of peptides **4a**, **5b**, **6a,** and **6b** were performed on a Ganesha 300XL system (Xenocs, France). Data were collected at room temperature over a Q range of 0.007 – 0.25 Å^−1^ for an exposure time of 3,600 s. All measurements were corrected for transmission and absolute intensity and had the solvent background and empty capillary scattering subtracted before processing. Data were reduced using SAXSGUI. Solutions prepared as described above were transferred to 80 × 2 mm (wall thickness 0.1 mm) quartz capillaries using a 1 mL syringe and 21 G needle and sealed using UV cured glue. A capillary with H_2_O was prepared and measured as a background.

The background was subtracted from the 1D plots in SasView 6.0,^57^ then the background corrected plots were fitted to structural models. The scattering length density (SLD) was calculated using the NIST neutron activation and scattering calculator (https://www.ncnr.nist.gov/resources/activation/), and the following SLD values were used: deionized H_2_O = 9.469 × 10^−6^ Å^−2^, peptide **4a** = 14.182 × 10^−6^ Å^−2^, peptide **4b** = 14.144 × 10^−6^ Å^−2^, peptide **5b** = 14.083 × 10^−6^ Å^−2^, peptide **6a** = 14.182 × 10^−6^ Å^−2^, and peptide **6b** = 14.144 × 10^−6^ Å^−2^.

### Cryo-EM

Solutions for Cryo-EM were prepared in volumes of at least 1 mL using the “slow addition” method described above. The sample was vitrified on lacey carbon grid using a Vitrobot Mark IV (Thermo Fisher Scientific). First, the surface of lacey carbon grid was made hydrophilic by glow-discharging it in the GloQube (Quorum Technologies). Then, ~3 μL of sample was applied on the glow-discharged grid, and excess sample was blotted away by Whatman filter paper (1001–055), leaving the thin film of sample on grid, which was plunge frozen in liquid ethane. The frozen grid was imaged on a 200 keV cryo-electron microscope (Glacios, Thermo Fisher Scientific) equipped with a cryo-autoloader, field emission gun (XFEG) electron source, and Falcon4 direct electron detector, housed in the University of Virginia Molecular Electron Microscopy Core facility.

### Preparation of heat-cooled two-component peptide solutions

Heat-cool solutions for CD measurements were carried out on an Anton Paar Physica MCR 302e rheometer equipped with a Huber RS232 PT100 water bath. Temperature sweeps were performed using a concentric cone geometry (CC27/T200/SS/GX) with a gap of 0.1 mm. Samples were heated using a linear temperature gradient from 25°C to 80°C over 90 min, held at 80°C for 30 min, then cooled to 25°C over 450 min and held at 25°C for 150 min. Measurements were taken using a fixed strain of 0.5% and angular frequency 1 rad s^−1^. A strain sweep from 0.1% to 100% with angular frequency of 1 rad s^−1^ was performed after the measurement. A portion of the resulting gel-like material was transferred to the cuvette for CD measurement.

### Fourier transform infrared measurements

Solutions for Fourier transform infrared (FTIR) were prepared in the standard method described above, using D_2_O as the solvent due to the prominence of H_2_O signals in solution FTIR. Solutions were pD adjusted using the relationship^58^:

pD=pH+0.4

FTIR measurements were performed on a Thermo Scientific Nicolet Apex iZ10 KBr FTIR spectrometer using an open aperture. Measurements were taken using a resolution of 4 cm^−1^ over a range of 1,800–1,550 cm^−1^, with 32 scans taken for each measurement. A D_2_O background was measured and subtracted from each sample.

## Supplementary Material

1

Supplemental information can be found online at https://doi.org/10.1016/j.xcrp.2026.103551.

## Figures and Tables

**Figure 1.**
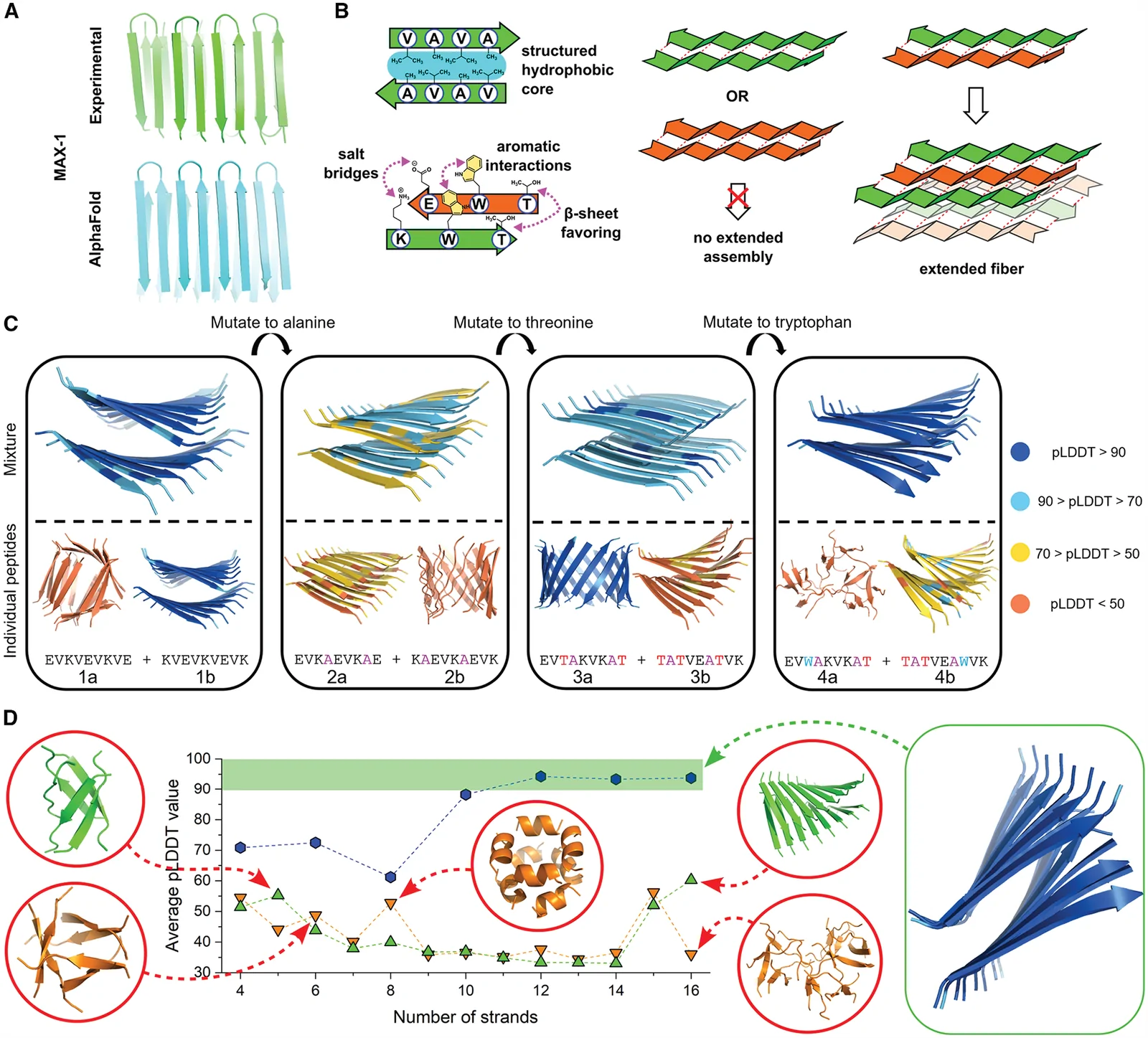
AlphaFold Multimer models predict capacity of designed peptides to self-assemble (A) Experimentally derived structure and AlphaFold2-predicted structure of MAX-1. See also Figure S1. (B) Schematic illustrating the intermolecular interactions targeted in the two-component structure, highlighting the promotion of extended co-assembled structures over assembly of each individual peptide. (C) AlphaFold structural predictions of four two-component peptide systems, illustrating the AlphaFold guided rational design process. The addition of alanine promotes no assembly of the individual peptide components, while adding β-promoting threonines and aromatic interactions improve confidence of the two-component fiber. See also Figures S3–S5. (D) A plot of number of peptide chains against average pLDDT value of the top ranked model for **4a**, **4b**, and **4a** + **4b**, where for the binary system, number of strands represents a total number of strands across the two peptides. Insets are AlphaFold2 structural predictions of **4a** (orange) and **4b** (green) across different numbers of strands. A prediction of **4a** + **4b** (16 strands), colored by pLDDT, is shown. See also Figure S6

**Figure 2.**
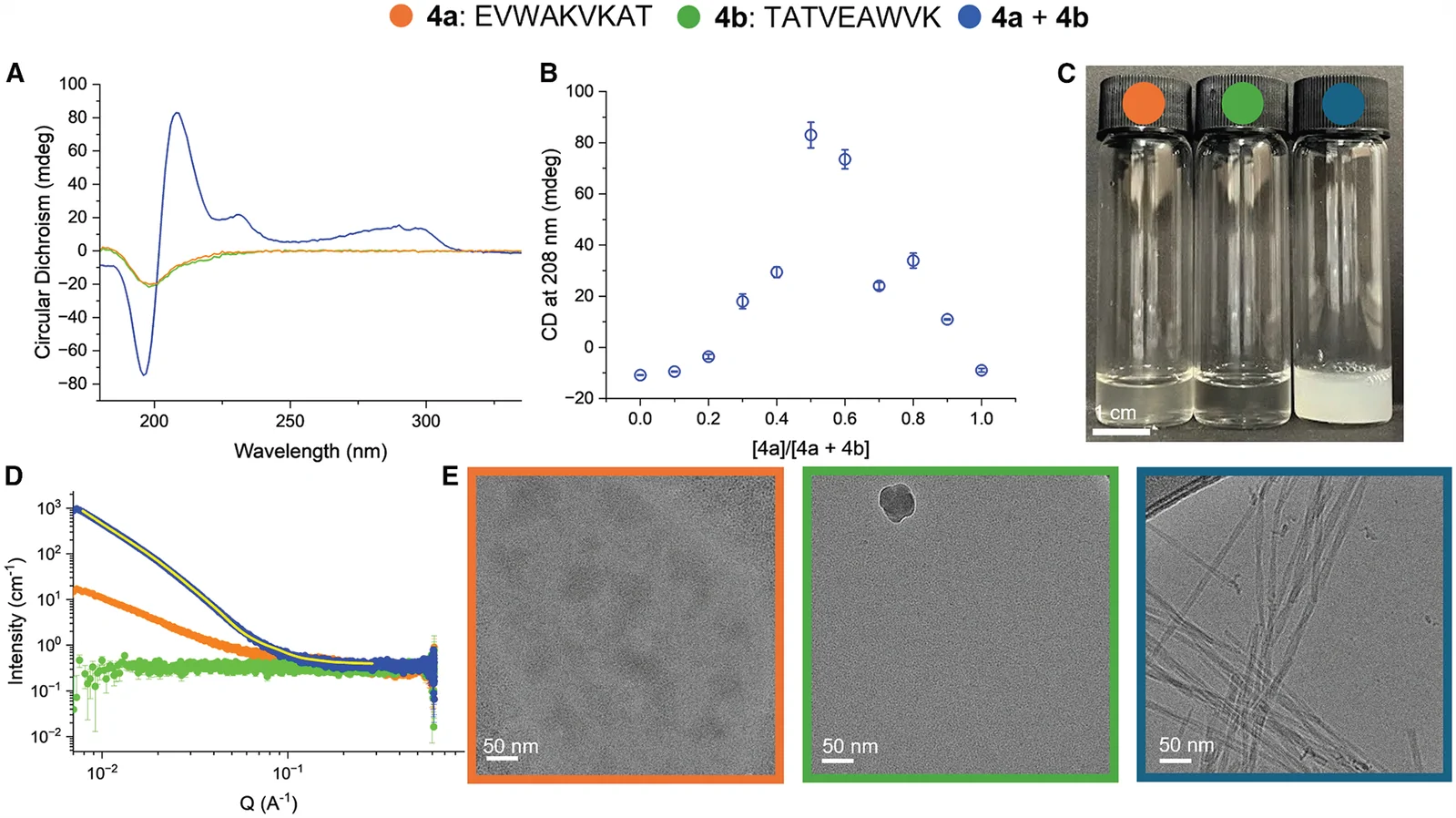
Experimental data for peptide system 4a + 4b (A) CD spectra of **4a** pH 7 10 mM (orange), **4b** pH 7 10 mM (green), **4a** + **4b** pH 7 5 + 5 mM (blue). (B) CD Job plot of **4a** + **4b** pH 7 at a total concentration of 10 mM. Data are represented as mean ± SEM. See also Figure S17. (C) Solutions of **4a** pH 7 10 mM (orange), **4b** pH 7 10 mM (green), and **4a** + **4b** pH 7 5 mM + 5 mM (blue). (D) SAXS data of **4a** pH 7 10 mM (orange), **4b** pH 7 10 mM (green), **4a** + **4b** pH 7 5 mM + 5 mM (blue), and fit (yellow line) of **4a** + **4b** pH 7 5 mM + 5 mM. The fit corresponds to an elliptical cylinder + power law model with radius 55 Å and axis ratio 2.5. See also Table S2. (E) Cryo-EM images of **4a** pH 7 10 mM (orange), **4b** pH 7 10 mM (green), and **4a** + **4b** pH 7 5 mM + 5 mM (blue). An ice crystal is pictured in the image of **4b**

**Figure 3.**
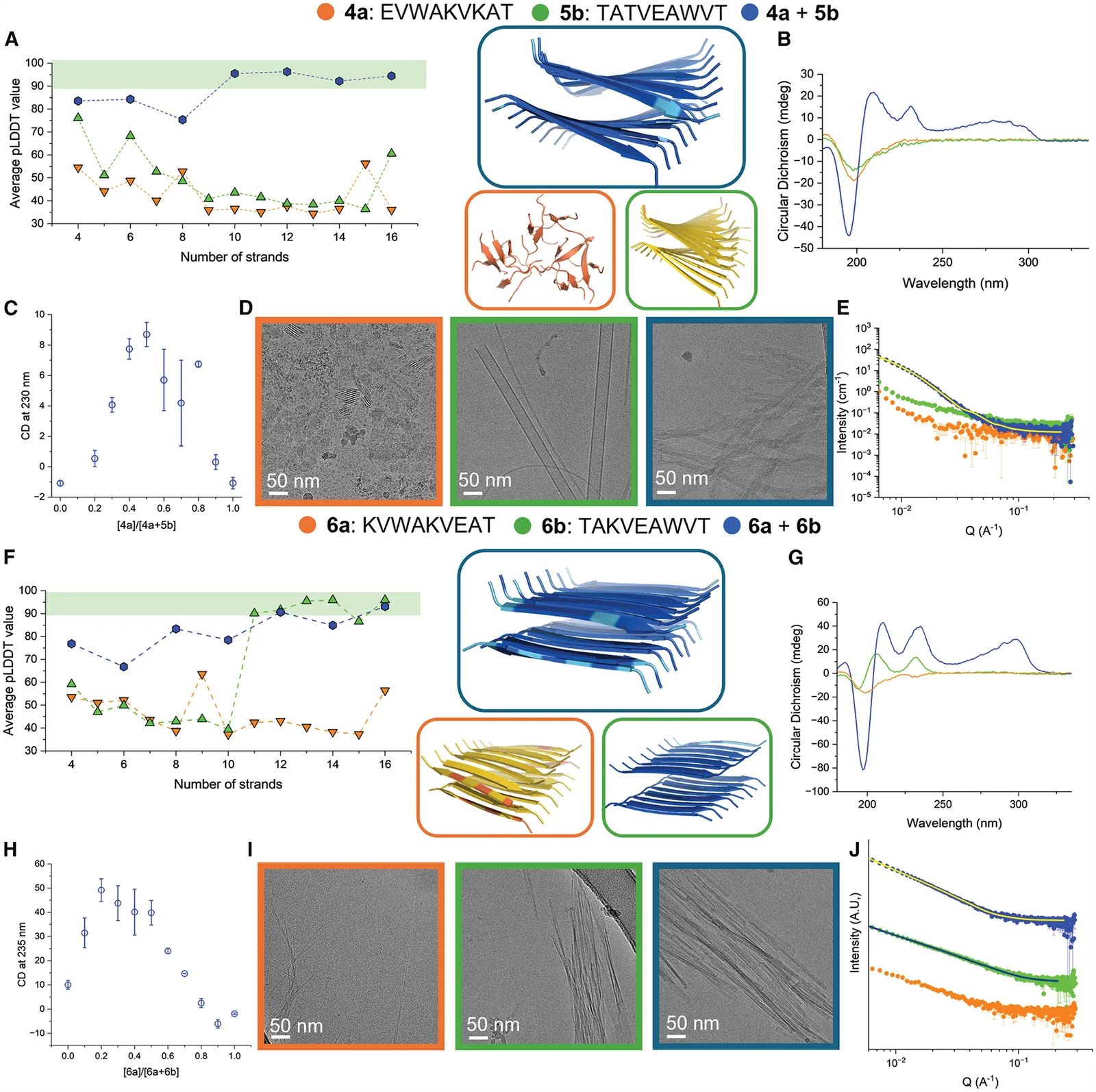
Similar peptide system design using AlphaFold (A) Plot of number of strands against average pLDDT value of the top model for **4a** (orange), **5b** (green), and **4a** + **5b** (blue), where the green shaded region corresponds to pLDDT >90. AlphaFold2 predicted structures, colored by pLDDT, of **4a** + **5b**, **4a**, and **5b** (16 strands) are shown. See also Figure S7. (B) CD spectra of **4a** pH 7 10 mM (orange), **5b** pH 7 10 mM (green), and **4a** + **5b** pH 7 5 mM + 5 mM (blue). (C) Job plot by CD of **4a** + **5b** pH 7 at a total concentration of 10 mM. Data are represented as mean ± SEM. As the CD signals of these solutions were lower intensity, small fluctuations in CD signal resulted in large error bars. See also Figure S20. (D) Cryo-EM images of **4a** pH 7 10 mM (orange), **5b** pH 7 10 mM (green), and **4a** + **5b** pH 7 5 mM + 5 mM (blue). (E) SAXS data of **4a** pH 7 10 mM (orange), **5b** pH 7 10 mM (green), **4a** + **5b** pH 7 5 mM + 5 mM (blue), and **4a** + **5b** pH 7 5 mM + 5 mM fit (yellow line). The data fit to an elliptical cylinder + power law model with radius 110 Å and axis ratio 2. See also Table S3. (F) Number of strands vs. pLDDT for **6a** (orange), **6b** (green), **6a** + **6b** (blue) where the green shaded region corresponds to pLDDT >90. AlphaFold2-predicted structures for **6a** + **6b**, **6a**, and **6b** (16 strands) are shown. See also Figure S8. (G) CD spectra of **6a** pH 7 10 mM (orange), **6b** pH 7 10 mM (green), and **6a** + **6b** pH 7 5 mM + 5 mM (blue). (H) Job plot by CD of **6a** + **6b** pH 7 at a total concentration of 10 mM. Data are represented as mean ± SEM. See also Figure S22. (I) Cryo-EM images of **6a** pH 7 10 mM (orange), **6b** pH 7 10 mM (green), and **6a** + **6b** pH 7 5 mM + 5 mM (blue). (J) SAXS data of **6a** pH 7 10 mM (orange), **6b** pH 7 10 mM (green), **6a** + **6b** pH 7 5 mM + 5 mM (blue), **6b** pH 7 10 mM fit (blue line), and **6a** + **6b** pH 7 5 mM + 5 mM fit (yellow line). The **6b** data fit to an elliptical cylinder + power law model with radius 25 Å and axis ratio 4. The **6a** + **6b** data fit to an elliptical cylinder + power law model with radius 55 Å and axis ratio 2.5. See also Tables S4 and S5

## Data Availability

Further information and requests for resources and reagents should be directed to the lead contact, Dave. J Adams (dave.adams@glasgow.ac.uk). All unique/stable reagents generated in this study are available from the lead contact upon request. All data are available in the main text or the supplemental information and deposited on Mendeley at https://doi.org/10.17632/hfzr4t853r.1. This paper does not report original code. Any additional information required to reanalyze the data reported in this paper is available from the lead contact upon request. Mendeley dataset preview: https://data.mendeley.com/preview/hfzr4t853r?a=650251c8-d297-4bb7-b580-4c2ae10b17d4.

## Highlights

- AlphaFold Multimer is used as a design tool to target conditional co-assembly
- Experimental data are consistent with the predicted tape-like structure
- AlphaFold can distinguish different behaviors between subtly different systems
