# Supplementary material for: Engineering conditional peptide self-assembly using AlphaFold
Source: Cell Rep Phys Sci. Author manuscript; Available in PMC 2026 Nov 6. (PMC13739434; doi:10.1016/j.xcrp.2026.103551)
Supplement: Supplementary file 1 [file NIHMS2218360-supplement-1.pdf]

Cell Reports Physical Science, Volume *7*

Supplemental information

Engineering conditional peptide self-assembly using AlphaFold

Helen Futty, Sofia Chinelli, Ravi R. Sonani, Tomás S. Plivelic, Annela Seddon, Edward H. Egelman, Andrew R. Thomson, and Dave J. Adams

Supplementary Figures

Computational results

All models produced in AlphaFold2 predictions were ranked by average predicted local distance difference test (pLDDT) score [S1]. The pLDDT is a score out of 100 of the local confidence of the positioning of each amino acid residue.

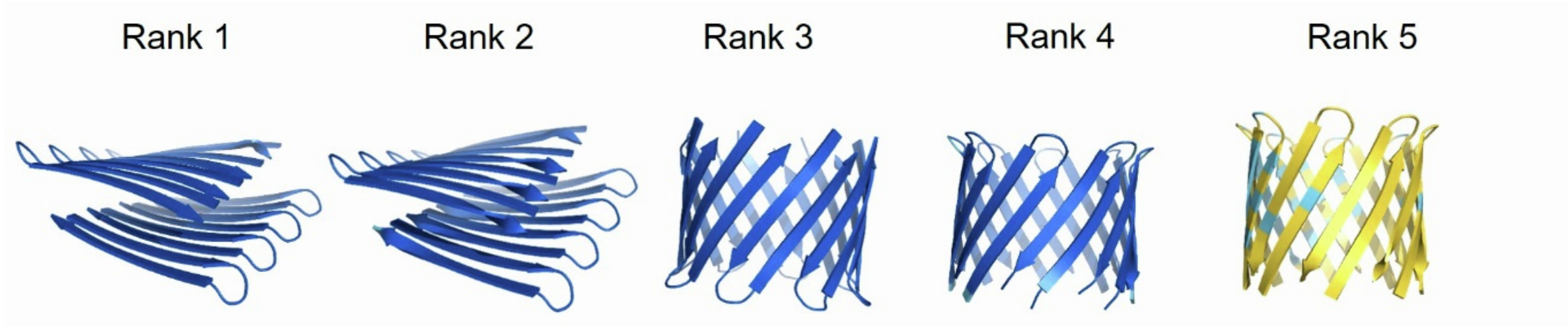

Supplementary Figure 1. Additional MAX-1 models. Related to Figure 1a. AlphaFold2 models rank 1-5 of MAX-1 (8 strands), coloured by pLDDT values. As AlphaFold2 can only run predictions of L-amino acids, the D-proline was substituted for a glycine residue, and the resulting amino acid sequence was VKVKVKVKGPTKVKVKVKV.

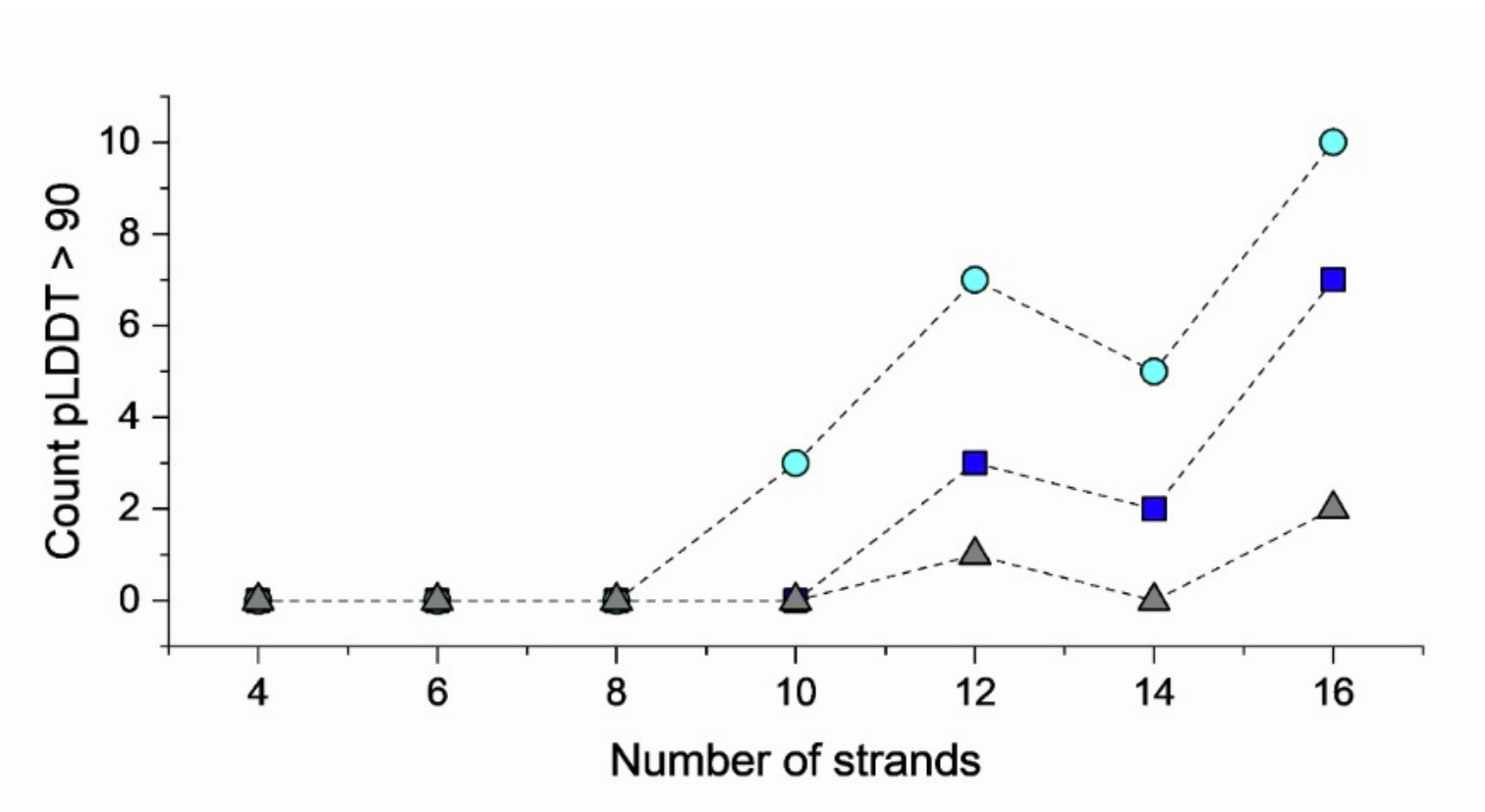

Supplementary Figure 2. Number of models with pLDDT > 90. Plot of number of strands used in a prediction against the number of models with average pLDDT value > 90 for **4a** + **4b** (blue), **4a** + **5b** (cyan), and **6a** + **6b** (grey).

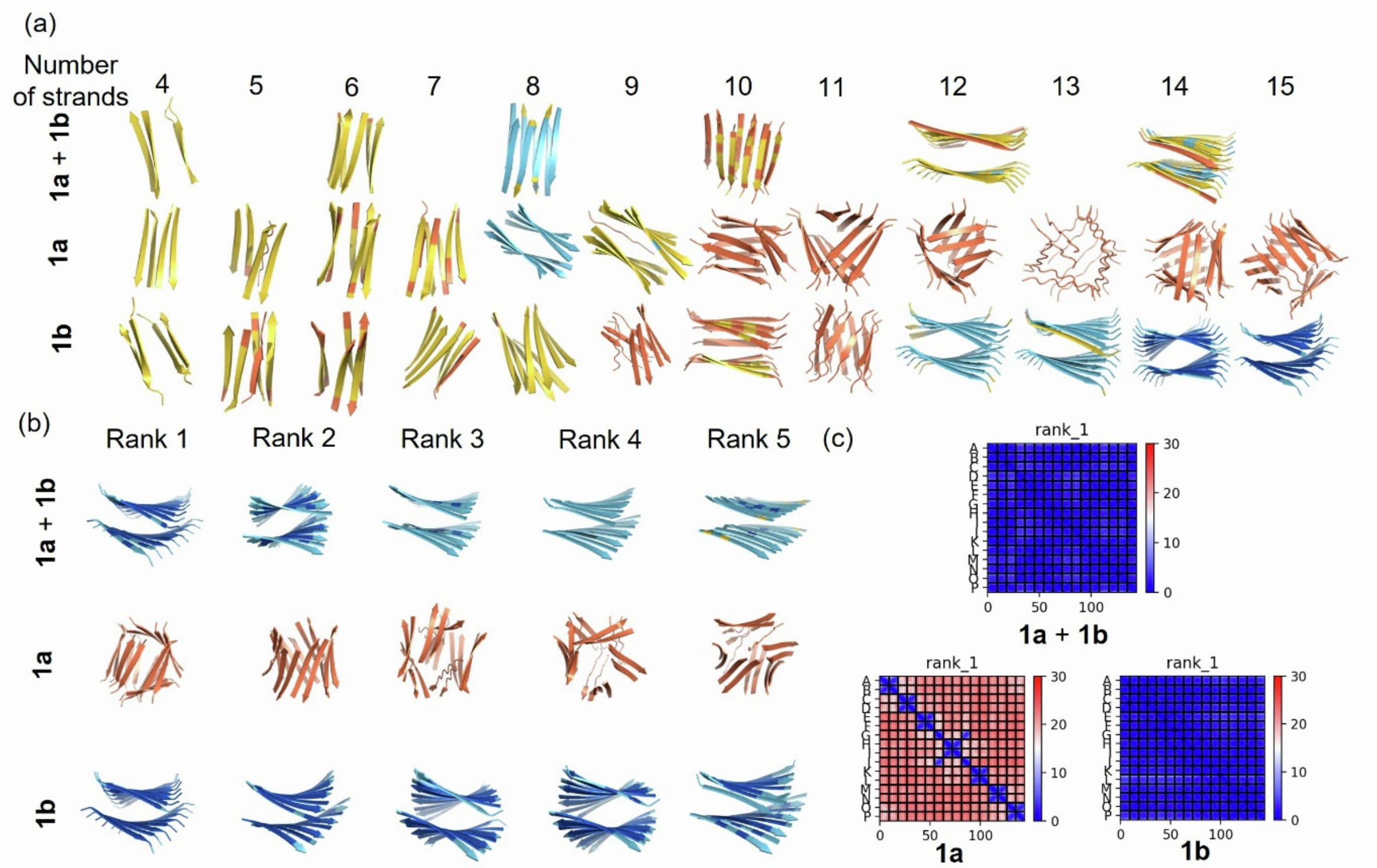

(d)

Peptide(s)	Average pLDDT	pTM	ipTM
**1a + 1b**	90.87	0.90	0.90
**1a**	38.75	0.17	0.13
**1b**	94.32	0.90	0.90

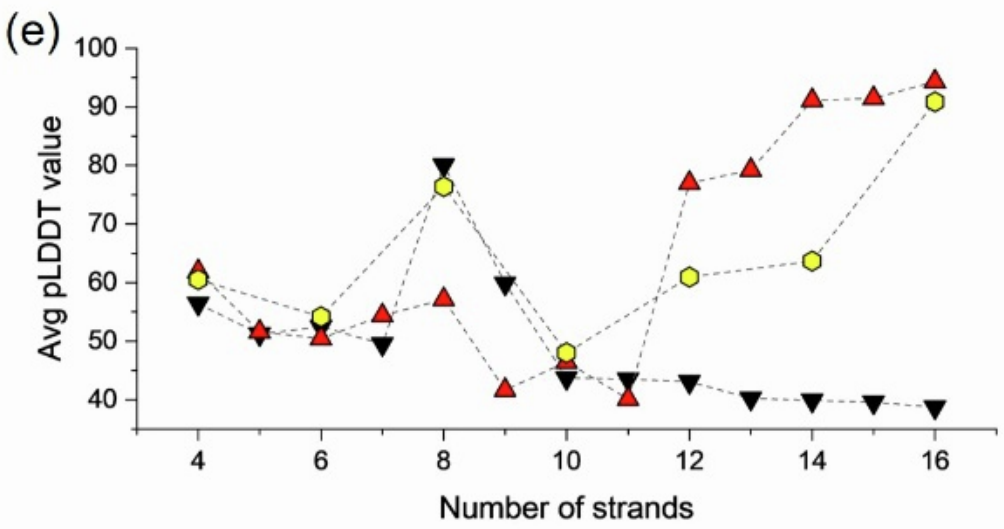

Supplementary Figure 3. AlphaFold models for peptide system 1. Related to Figure 1c. (a) AlphaFold2 rank 1 models of **1a** + **1b**, **1a**, and **1b** when 4 up to 15 strands are used in a prediction. Strand counts in the two-component system represent a total number of peptide chains, where an equal number of strands of **1a** and **1b** were used. For this reason, two-component predictions were carried out for even numbers of strands only. Models are coloured by pLDDT values. (b) AlphaFold2 models rank 1-5 of **1a** + **1b**, **1a**, and **1b** when 16 strands are used in the prediction. Models are coloured by pLDDT values. (c) Predicted aligned error plots for **1a** + **1b**, **1a**, and **1b**, where 16 strands are used in the prediction. (d) A table presenting the average pLDDT value, pTM and ipTM score of the top ranked model when 16 strands are used in the prediction. (e) Plot of number of strands used in a prediction versus the average pLDDT value of the highest ranked model for **1a** (black), **1b** (red) and **1a** + **1b** (yellow).

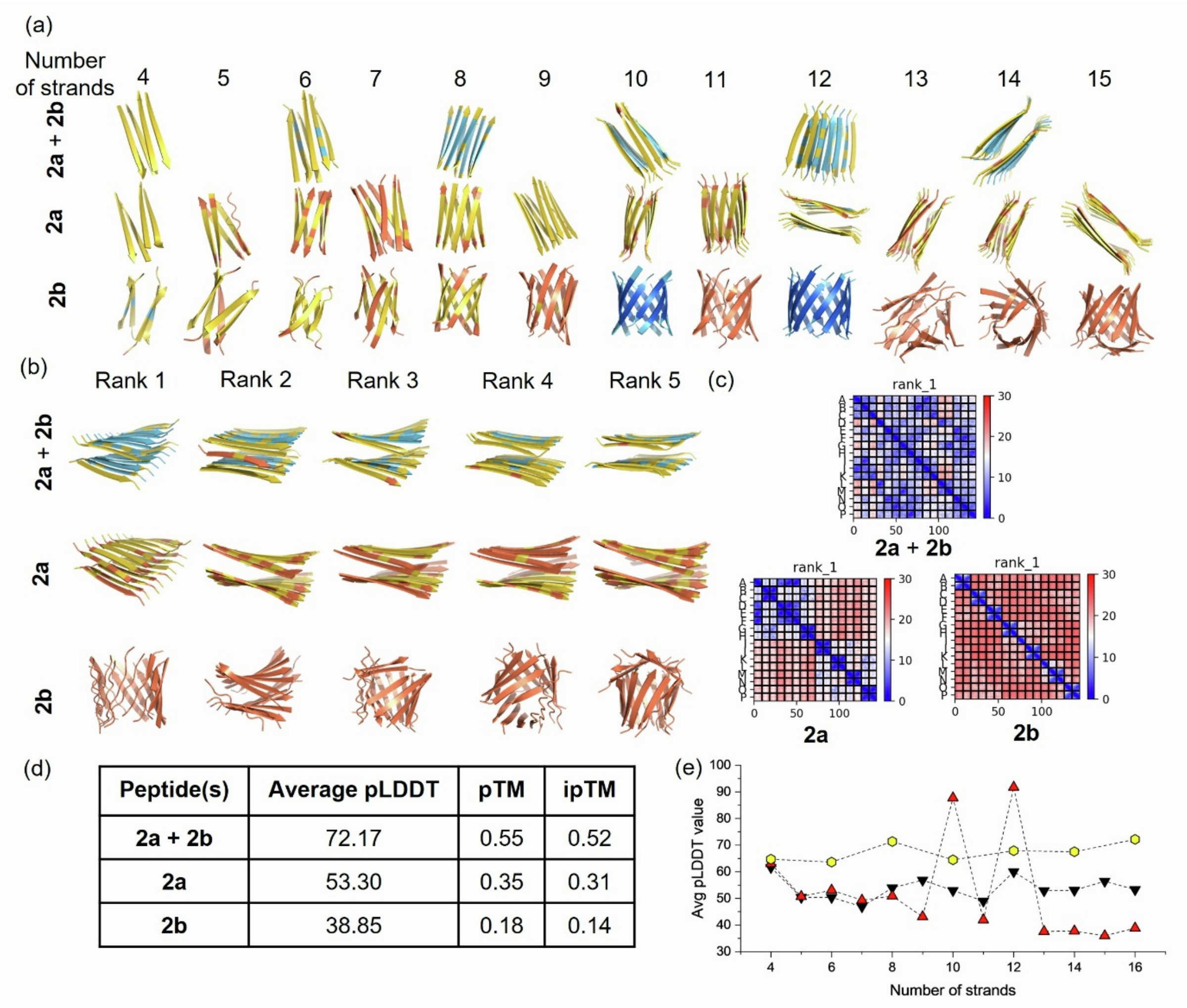

Peptide(s)	Average pLDDT	pTM	ipTM
**2a + 2b**	72.17	0.55	0.52
**2a**	53.30	0.35	0.31
**2b**	38.85	0.18	0.14

Supplementary Figure 4. AlphaFold models for peptide system 2. Related to Figure 1c. (a) AlphaFold2 rank 1 models of **2a** + **2b**, **2a**, and **2b** when 4 up to 15 strands are used in a prediction. Strand counts in the two-component system represent a total number of peptide chains, where an equal number of strands of **2a** and **2b** were used. For this reason, two-component predictions were carried out for even numbers of strands only. Models are coloured by pLDDT values. (b) AlphaFold2 models rank 1-5 of **2a** + **2b**, **2a**, and **2b** when 16 strands are used in the prediction. Models are coloured by pLDDT values. (c) Predicted aligned error plots for **2a** + **2b**, **2a**, and **2b**, when 16 strands are used in the prediction. (d) A table presenting the average pLDDT value, pTM and ipTM score of the top ranked model when 16 strands are used in the prediction. (e) Plot of number of strands used in a prediction versus the average pLDDT value of the highest ranked model for **2a** (black), **2b** (red) and **2a** + **2b** (yellow).

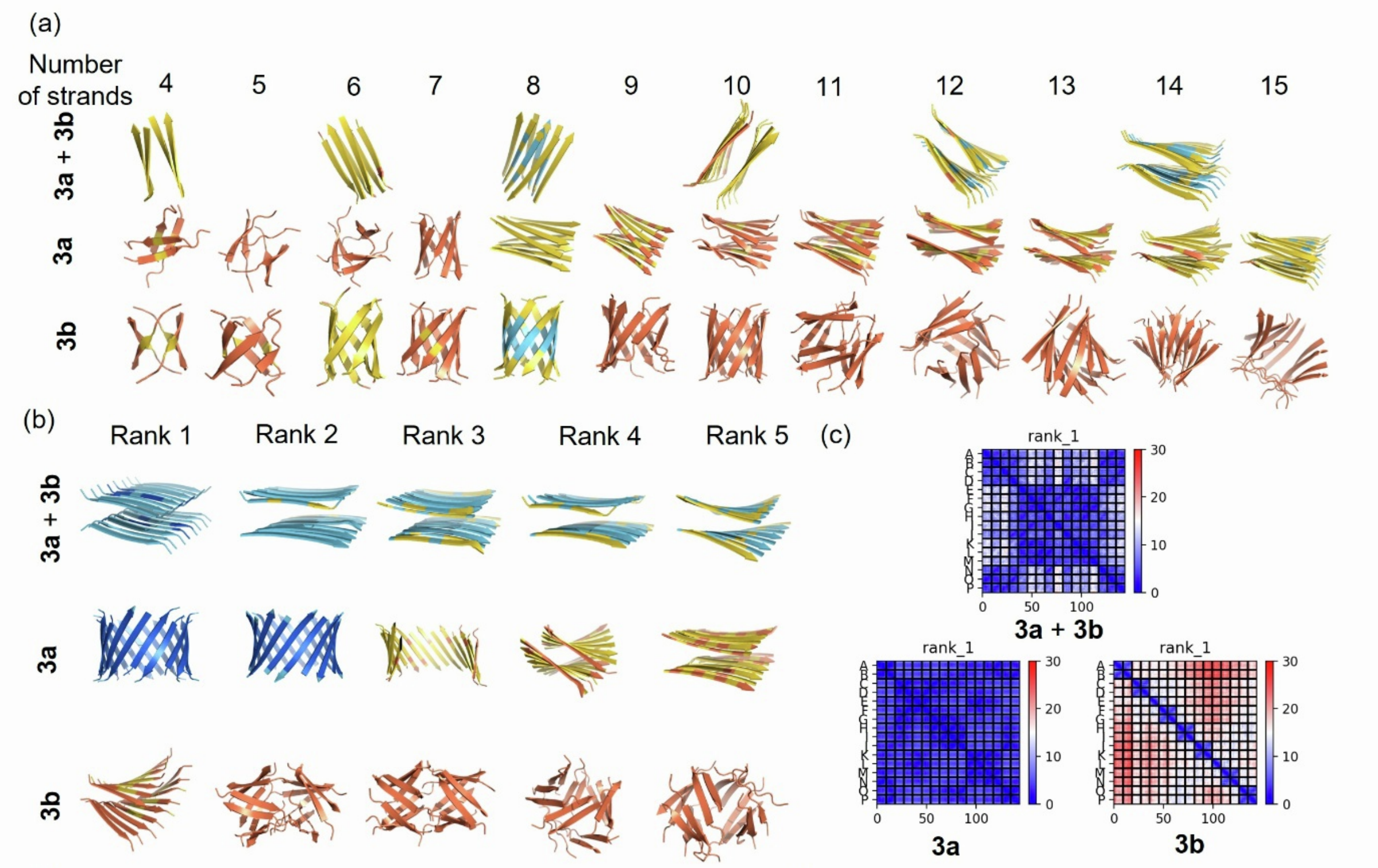

(d)

Peptide(s)	Average pLDDT	pTM	ipTM
3a + 3b	84.50	0.73	0.71
3a	92.52	0.76	0.75
3b	44.94	0.28	0.24

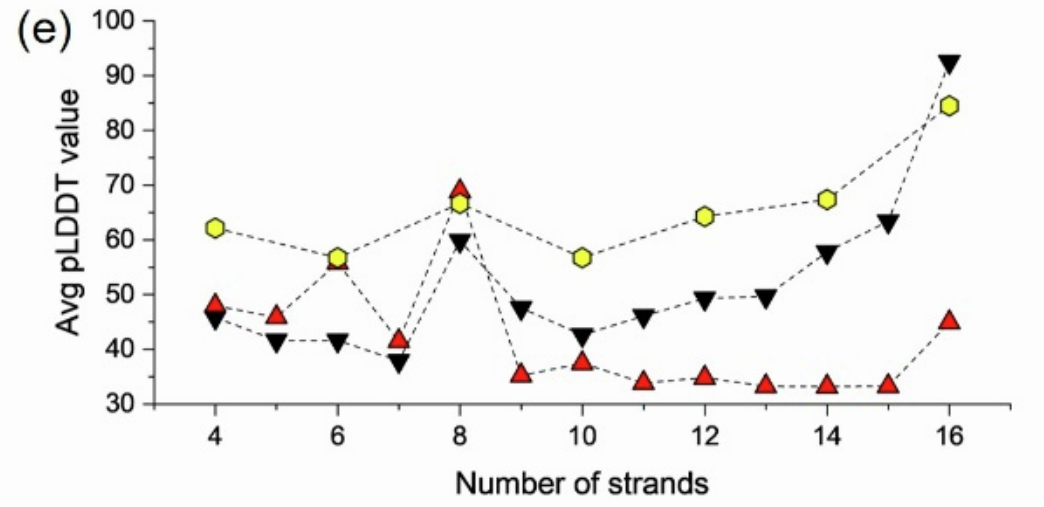

Supplementary Figure 5. AlphaFold models for peptide system 3. Related to Figure 1c. (a) AlphaFold2 rank 1 models of **3a** + **3b**, **3a**, and **3b** when 4 up to 15 strands are used in a prediction. Strand counts in the two-component system represent a total number of peptide chains, where an equal number of strands of **3a** and **3b** were used. For this reason, two-component predictions were carried out for even numbers of strands only. Models are coloured by pLDDT values. (b) AlphaFold2 models rank 1-5 of **3a** + **3b**, **3a**, and **3b** when 16 strands are used in the prediction. Models are coloured by pLDDT values. (c) Predicted aligned error plots for **3a** + **3b**, **3a**, and **3b**, when 16 strands are used in the prediction. (d) A table presenting the average pLDDT value, pTM and ipTM score of the top ranked model when 16 strands are used in the prediction. (e) Plot of number of strands used in a prediction versus the average pLDDT value of the highest ranked model for **3a** (black), **3b** (red) and **3a** + **3b** (yellow).

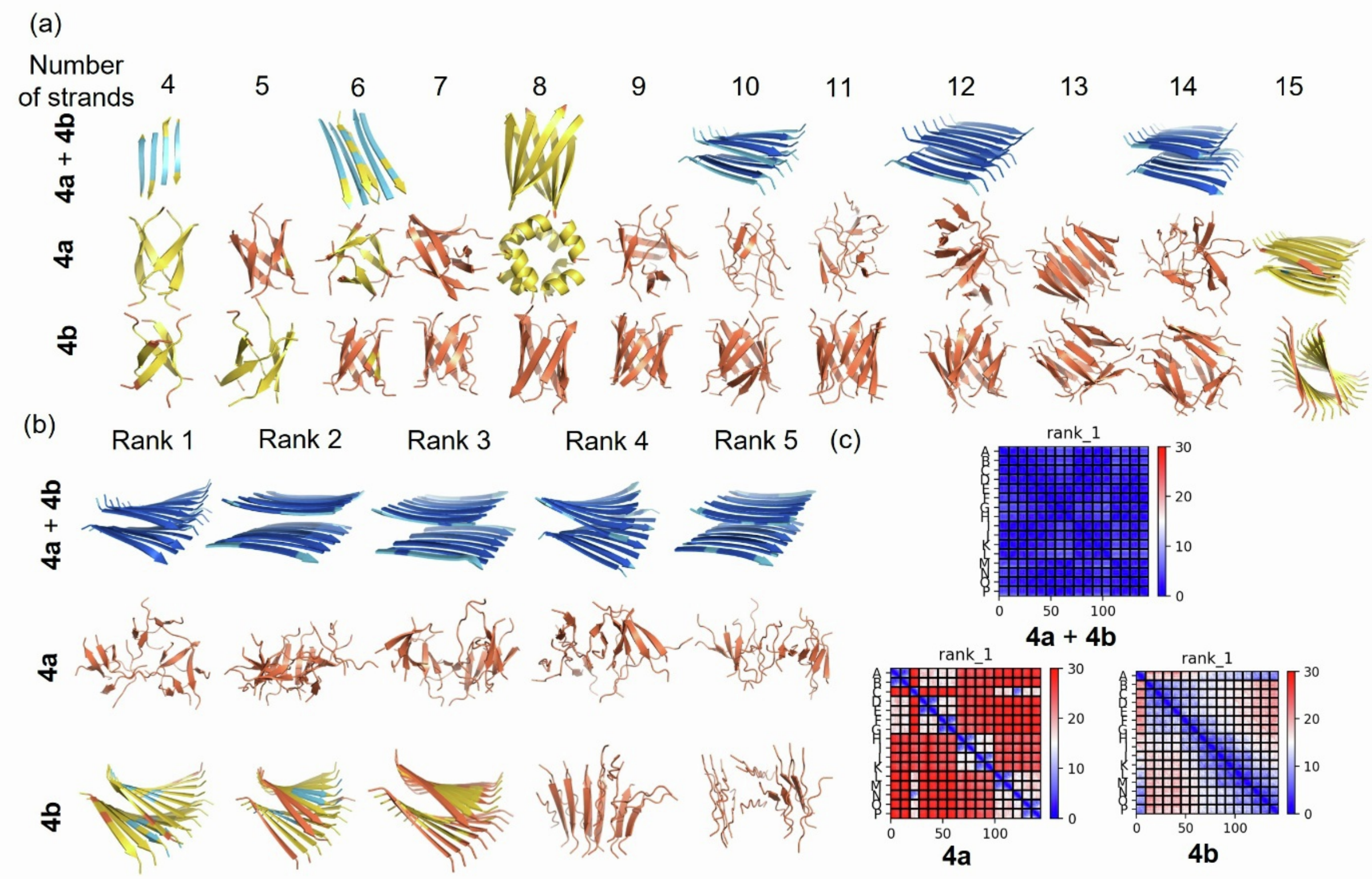

(d)

Peptide(s)	Average pLDDT	pTM	ipTM
**4a + 4b**	93.76	0.86	0.86
**4a**	36.03	0.17	0.13
**4b**	60.24	0.48	0.46

Supplementary Figure 6. AlphaFold models for peptide system 4. Related to Figure 1d. (a) AlphaFold2 rank 1 models of **4a** + **4b**, **4a**, and **4b** when 4 up to 15 strands are used in a prediction. Strand counts in the two-component system represent a total number of peptide chains, where an equal number of strands of **4a** and **4b** were used. For this reason, two-component predictions were carried out for even numbers of strands only. Models are coloured by pLDDT values. (b) AlphaFold2 models rank 1-5 of **4a** + **4b**, **4a**, and **4b** when 16 strands are used in the prediction. Models are coloured by pLDDT values. (c) Predicted aligned error plots for **4a** + **4b**, **4a**, and **4b**, when 16 strands are used in the prediction. (d) A table presenting the average pLDDT value, pTM and ipTM score of the top ranked model when 16 strands are used in the prediction.

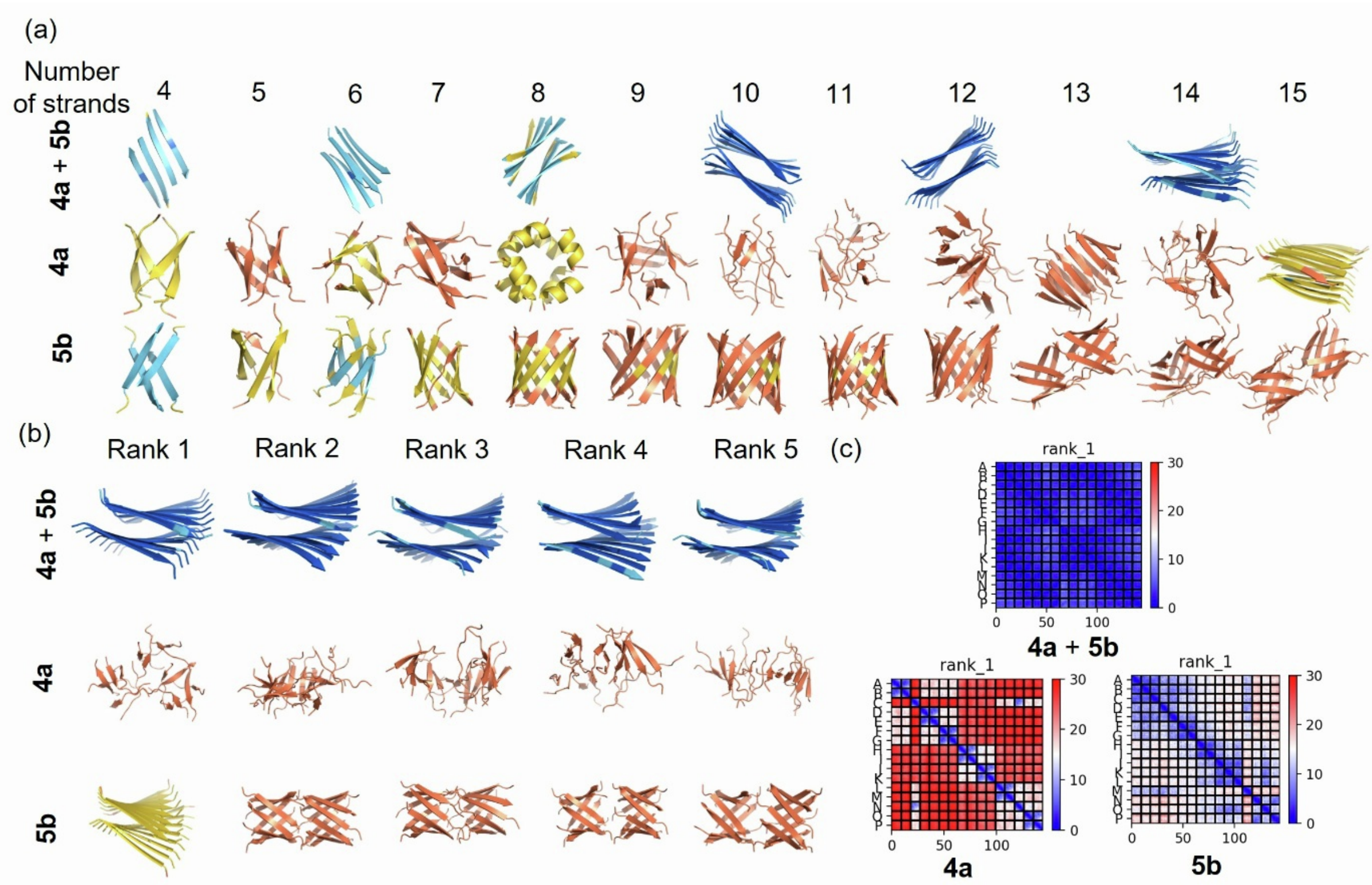

(d)

Peptide(s)	Average pLDDT	pTM	ipTM
**4a + 5b**	94.44	0.87	0.86
**4a**	36.03	0.17	0.13
**5b**	60.57	0.49	0.46

Supplementary Figure 7. AlphaFold models for peptide system 5. Related to Figure 3a. (a) AlphaFold2 rank 1 models of **4a** + **5b**, **4a**, and **5b** when 4 up to 15 strands are used in a prediction. Strand counts in the two-component system represent a total number of peptide chains, where an equal number of strands of **4a** and **5b** were used. For this reason, two-component predictions were carried out for even numbers of strands only. Models are coloured by pLDDT values. (b) AlphaFold2 models rank 1-5 of **4a** + **5b**, **4a**, and **5b** when 16 strands are used in the prediction. Models are coloured by pLDDT values. (c) Predicted aligned error plots for **4a** + **5b**, **4a**, and **5b**, when 16 strands are used in the prediction. (d) A table presenting the average pLDDT value, pTM and ipTM score of the top ranked model when 16 strands are used in the prediction.

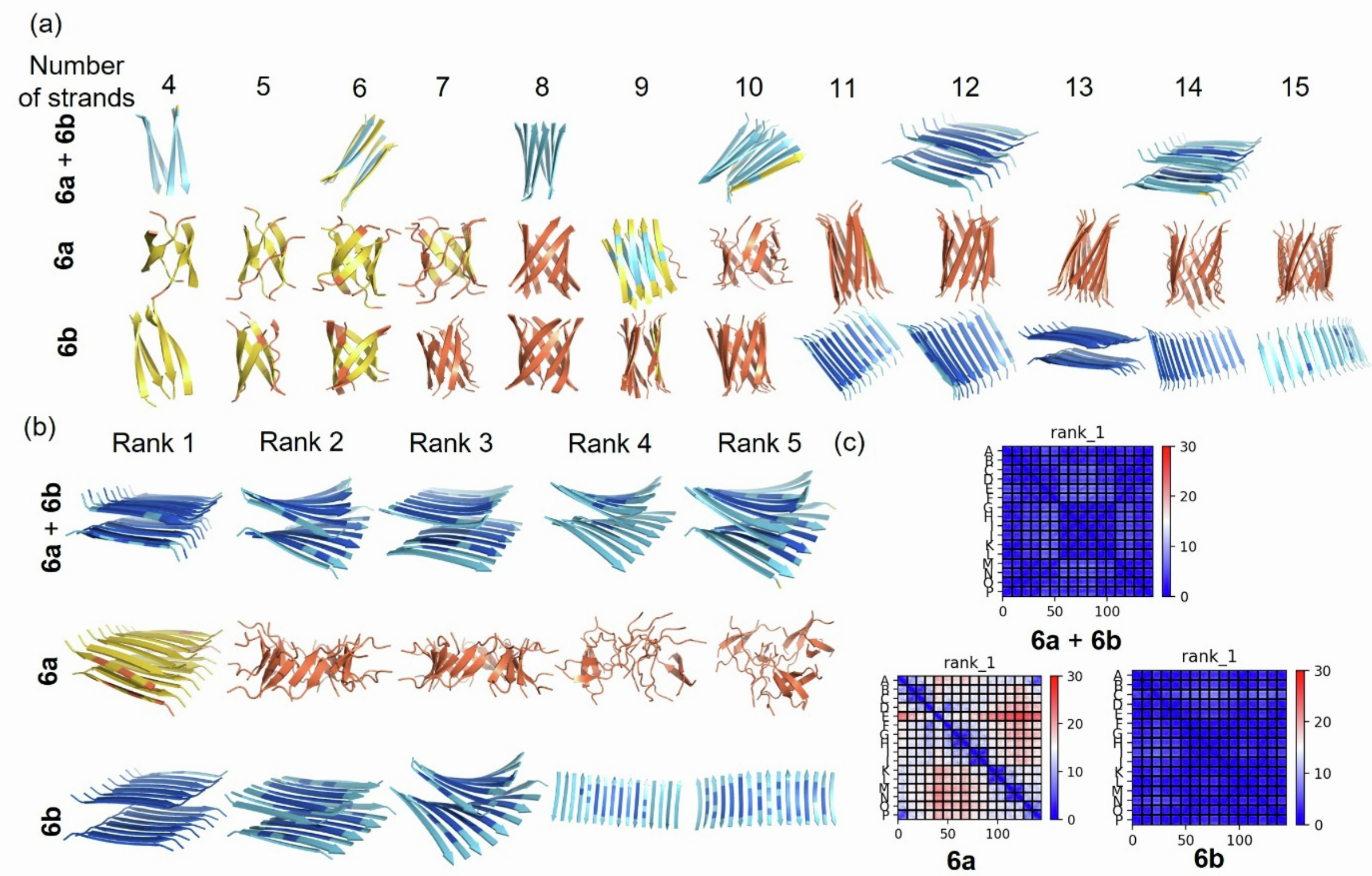

(d)

Peptide(s)	Average pLDDT	pTM	ipTM
**6a + 6b**	93.07	0.85	0.85
**6a**	56.41	0.37	0.34
**6b**	95.91	0.88	0.88

Supplementary Figure 8. AlphaFold models for peptide system 6. Related to Figure 3f. (a) AlphaFold2 rank 1 models of **6a** + **6b**, **6a**, and **6b** when 4 up to 15 strands are used in a prediction. Strand counts in the two-component system represent a total number of peptide chains, where an equal number of strands of **6a** and **6b** were used. For this reason, two-component predictions were carried out for even numbers of strands only. Models are coloured by pLDDT values. (b) AlphaFold2 models rank 1-5 of **6a** + **6b**, **6a**, and **6b** when 16 strands are used in the prediction. Models are coloured by pLDDT values. (c) Predicted aligned error plots for **6a** + **6b**, **6a**, and **6b**, when 16 strands are used in the prediction. (d) A table presenting the average pLDDT value, pTM and ipTM score of the top ranked model when 16 strands are used in the prediction.

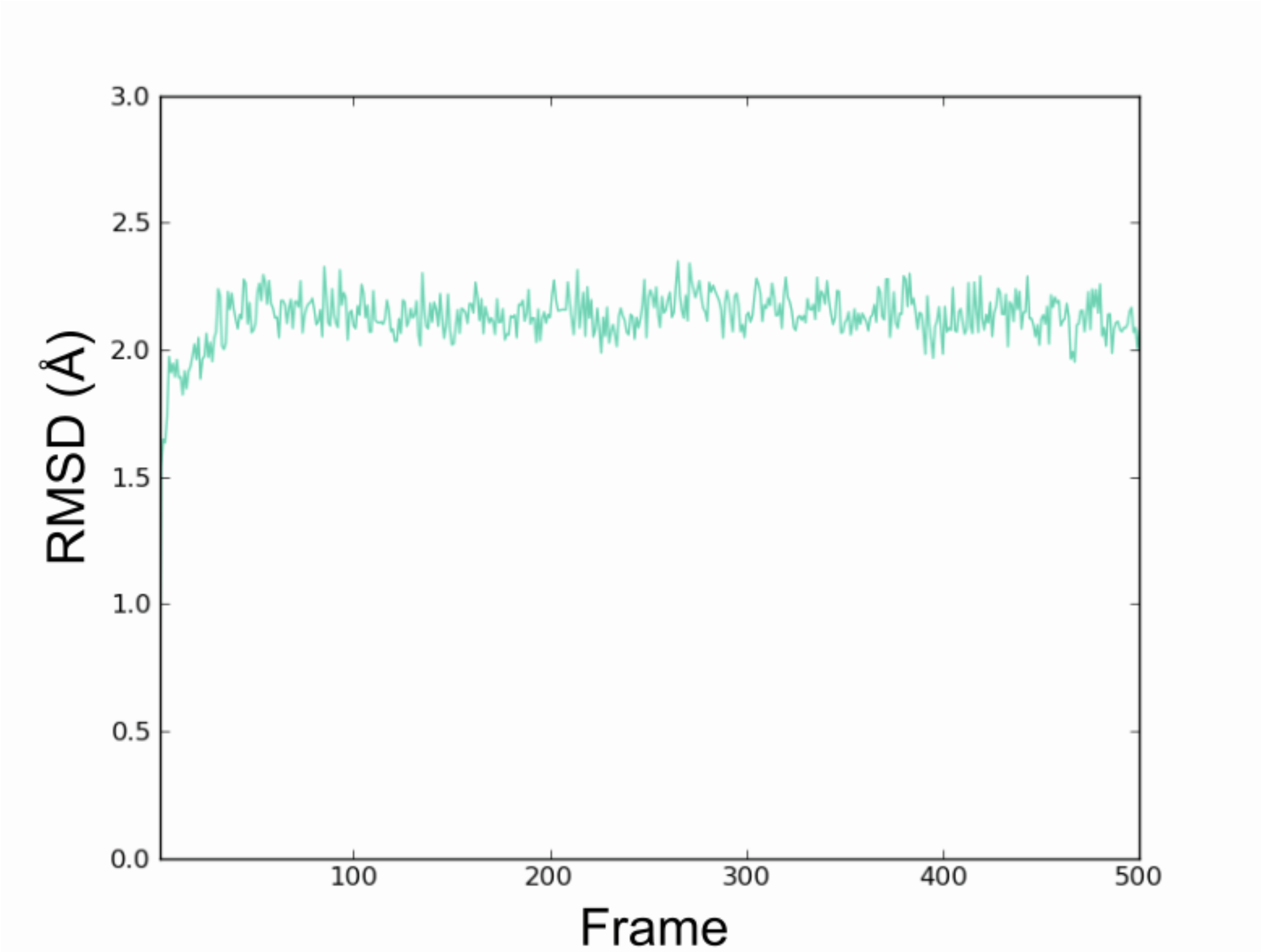

Supplementary Figure 9. Molecular dynamics simulation of 4a + 4b. RMSD value for 100ns MD trajectory of the **4a** + **4b** double layered structure. Frames were sampled evenly across the 100ns trajectory. MD was run using openMM and using Amber99 and tip3p water, with periodic boundary conditions. Timesteps were 2fs. Sodium and chloride ions were added to mimic an ionic strength of 0.1M. The start temperature was 350K, cooling linearly to 300K over the first 50ns, and 300K thereafter.

Peptide purity analysis

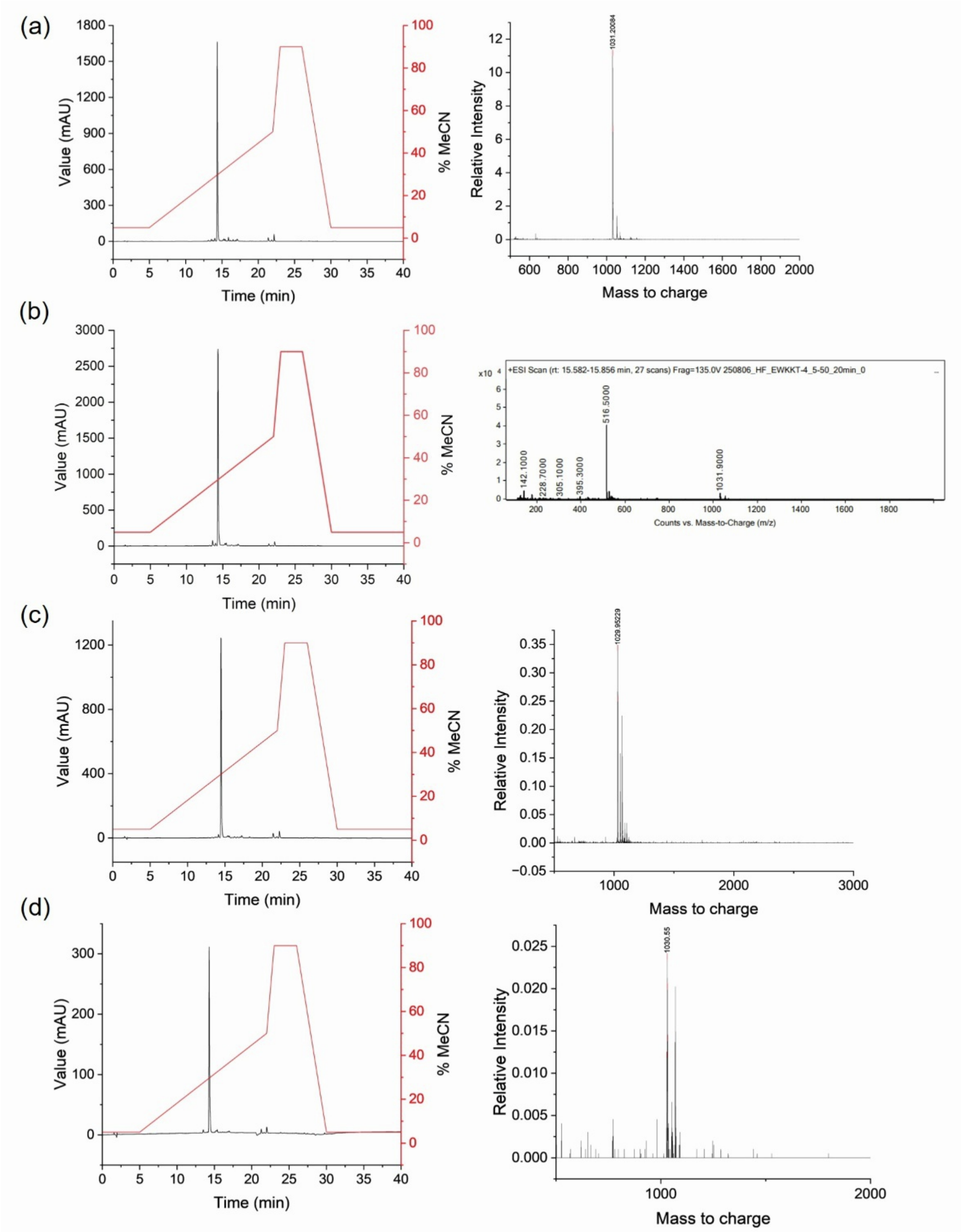

Supplementary Figure 10. Purity of peptide 4a. HP-LC chromatogram and mass spectrometry of four **4a** batches used. The purity was calculated to be greater than 90 % using a 20-minute gradient elution of 5-50 % MeCN in H_2O. The peptide has mass 1031.21 Da, and this was confirmed by MALDI-TOF spectrometry in cases (a), (c) and (d). Peptide (b) mass was confirmed by LC-MS using a 20-minute gradient elution of 5-50 % MeCN in H_2O.

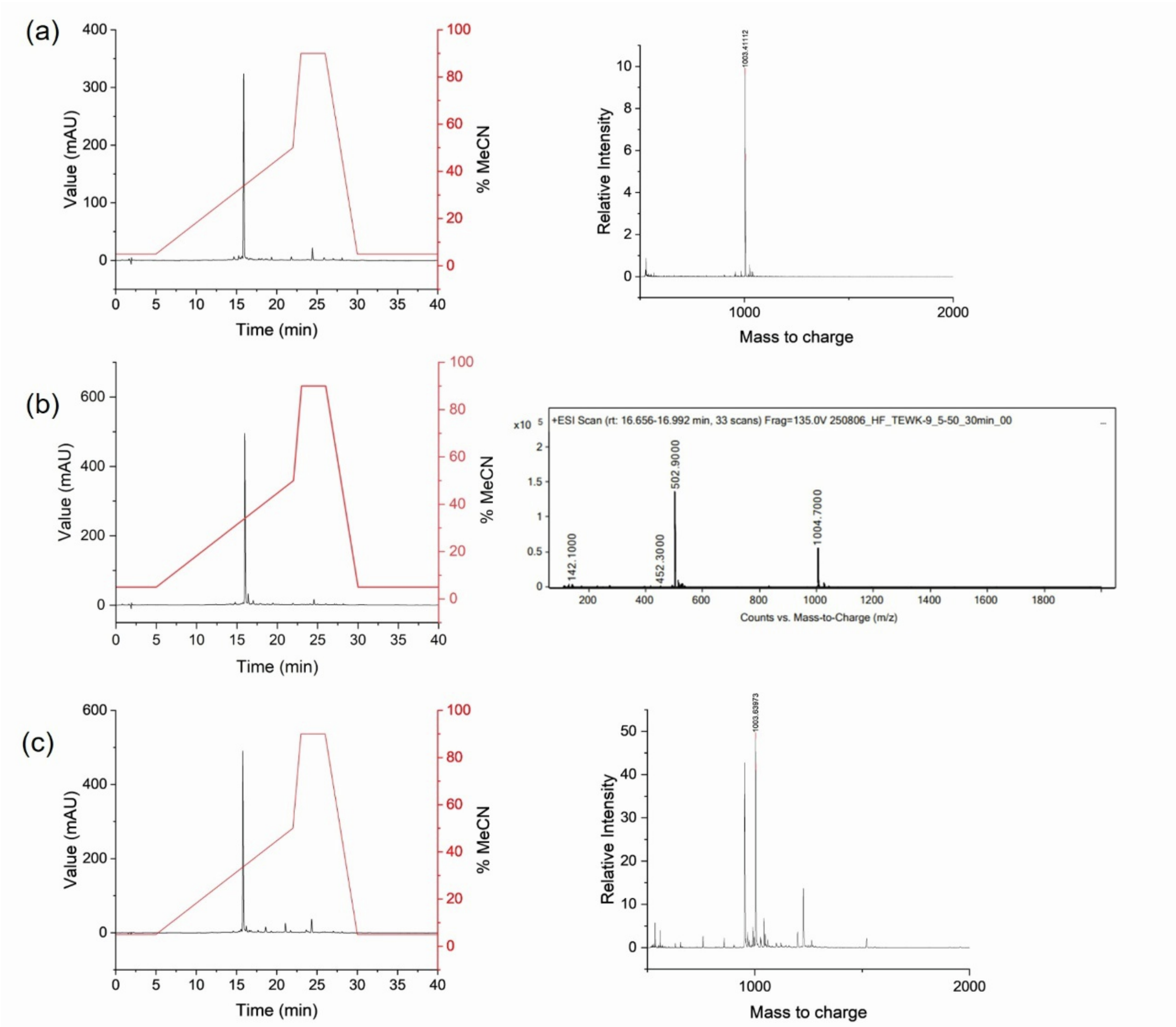

Supplementary Figure 11. Purity of peptide 4b. HP-LC chromatogram and mass spectrometry of three **4b** batches used. The purity was calculated to be greater than 90 % using a 20-minute gradient elution of 5-50 % MeCN in H_2O. The peptide has mass 1004.14 Da, and this was confirmed by MALDI-TOF spectrometry in cases (a) and (c). Peptide (b) mass was confirmed by LC-MS using a 20-minute gradient elution of 5-50 % MeCN in H_2O.

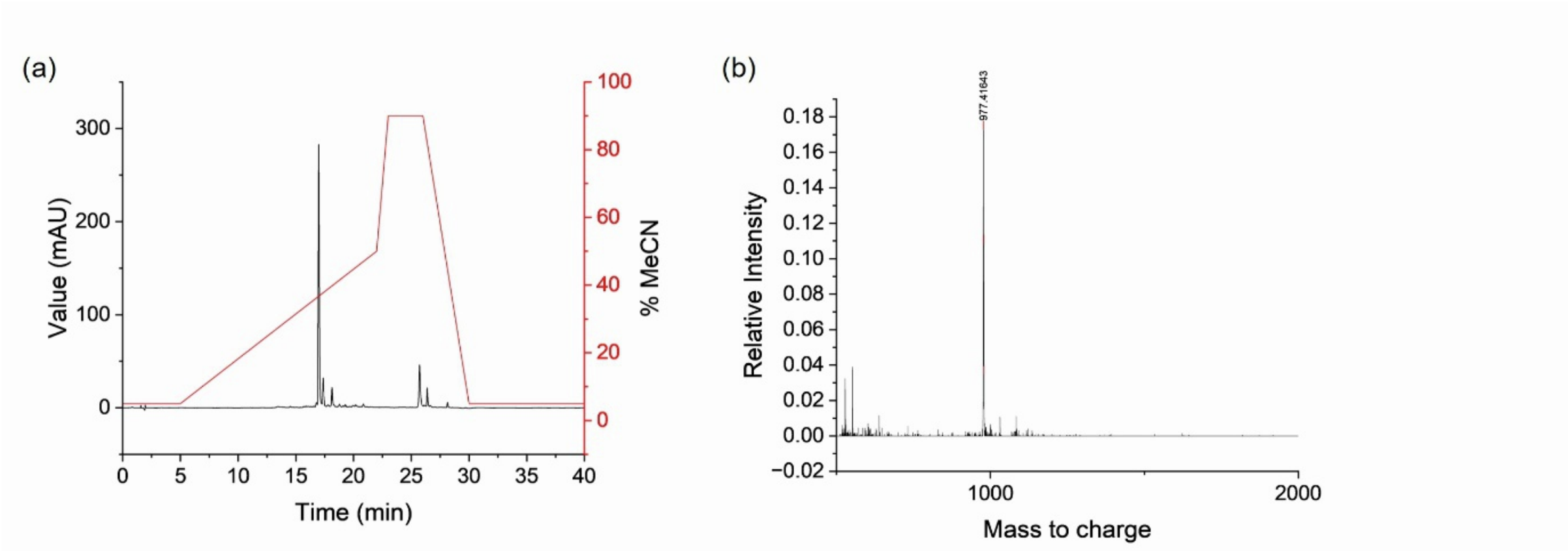

Supplementary Figure 12. Purity of peptide 5b. (a) HP-LC chromatogram and (b) mass spectrometry of **5b**. The purity was calculated to be approximately 80 % using a 20-minute gradient elution of 5-50 % MeCN in H_2O. The peptide has mass 977.07 Da, and this was confirmed by MALDI-TOF spectrometry.

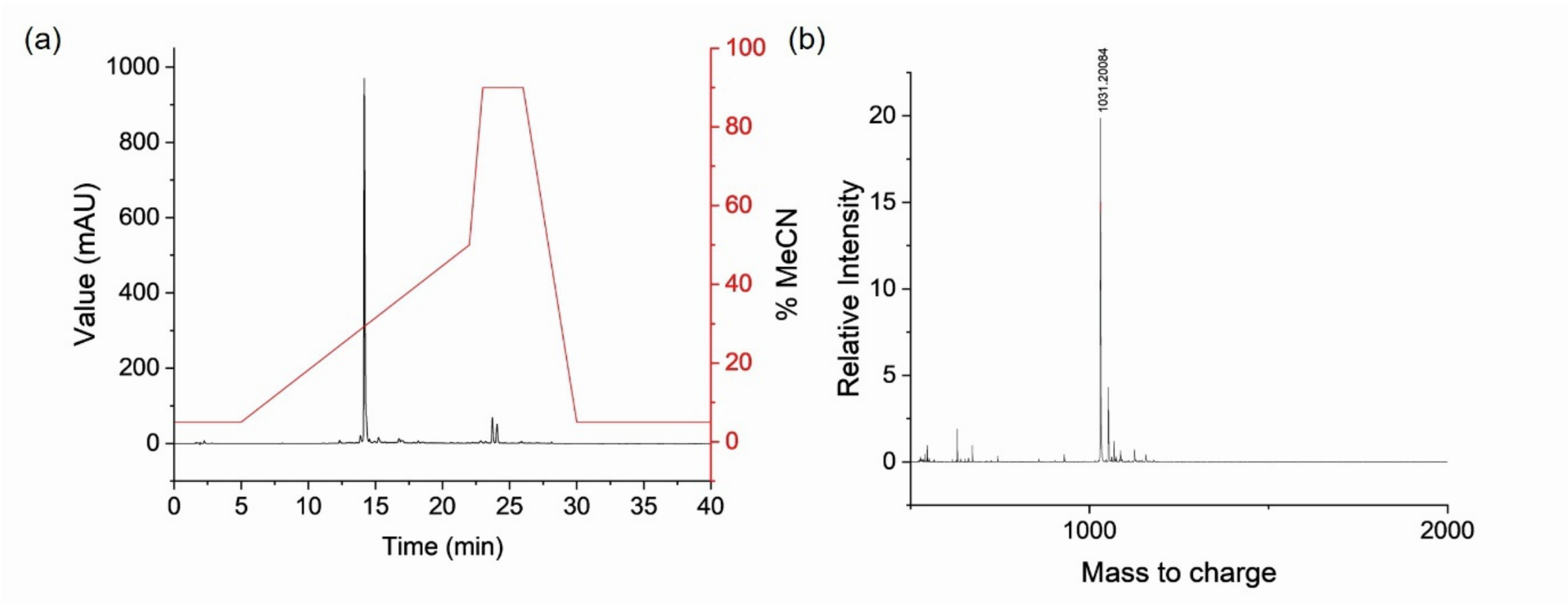

Supplementary Figure 13. Purity of peptide 6a. (a) HP-LC chromatogram and (b) mass spectrometry of **6a**. The purity was calculated to be greater than 90 % using a 20-minute gradient elution of 5-50 % MeCN in H_2O. The peptide has mass 1031.21 Da, and this was confirmed by MALDI-TOF spectrometry.

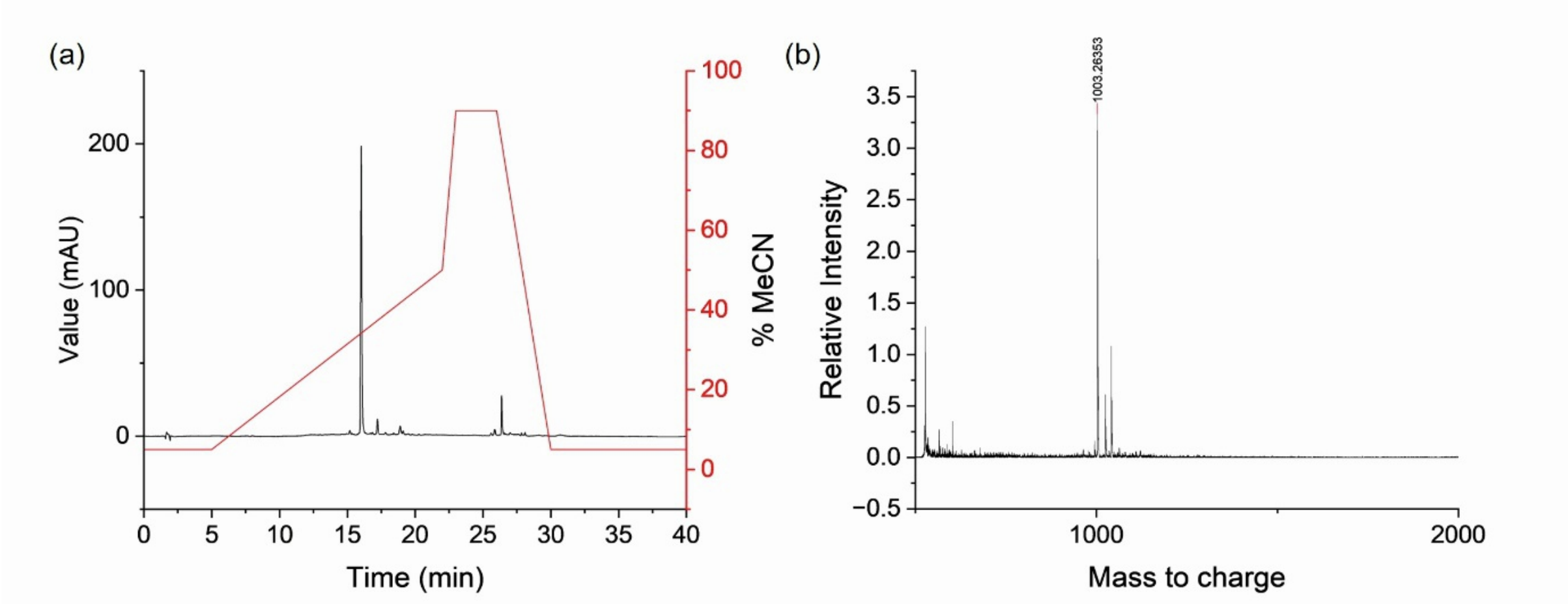

Supplementary Figure 14. Purity of peptide 6b. (a) HP-LC chromatogram and (b) mass spectrometry of **6b**. The purity was calculated to be greater than 90 % using a 20-minute gradient elution of 5-50 % MeCN in H_2O. The peptide has mass 1004.14 Da, and this was confirmed by MALDI-TOF spectrometry.

Circular dichroism

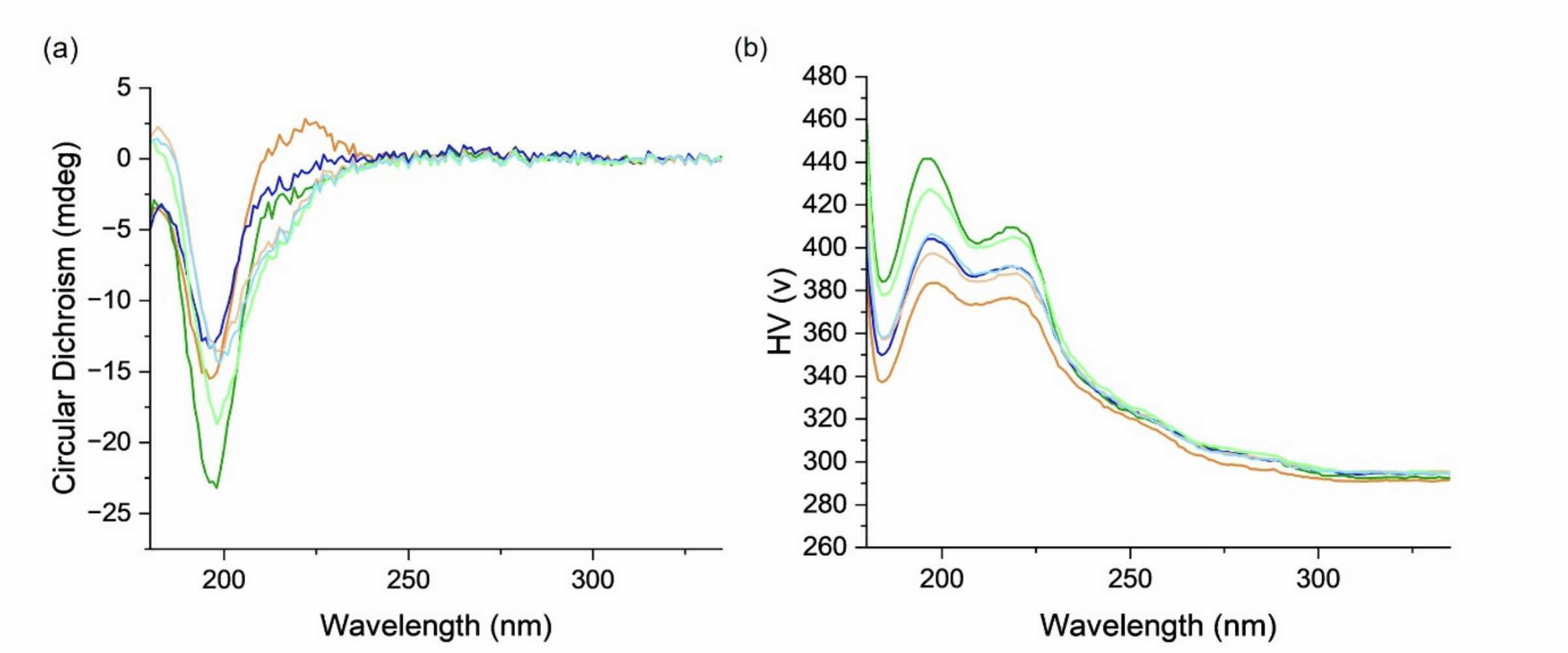

Supplementary Figure 15. CD at extreme pH of 4a + 4b. (a) CD and (b) HV data of **4a** pH 2 10 mM (dark orange), **4b** pH 2 10 mM (dark green), **4a** + **4b** pH 2 5 mM + 5 mM (dark blue), **4a** pH 12 10 mM (light orange), **4b** pH 12 10 mM (light green) and **4a** + **4b** pH 12 5 mM + 5 mM (light blue).

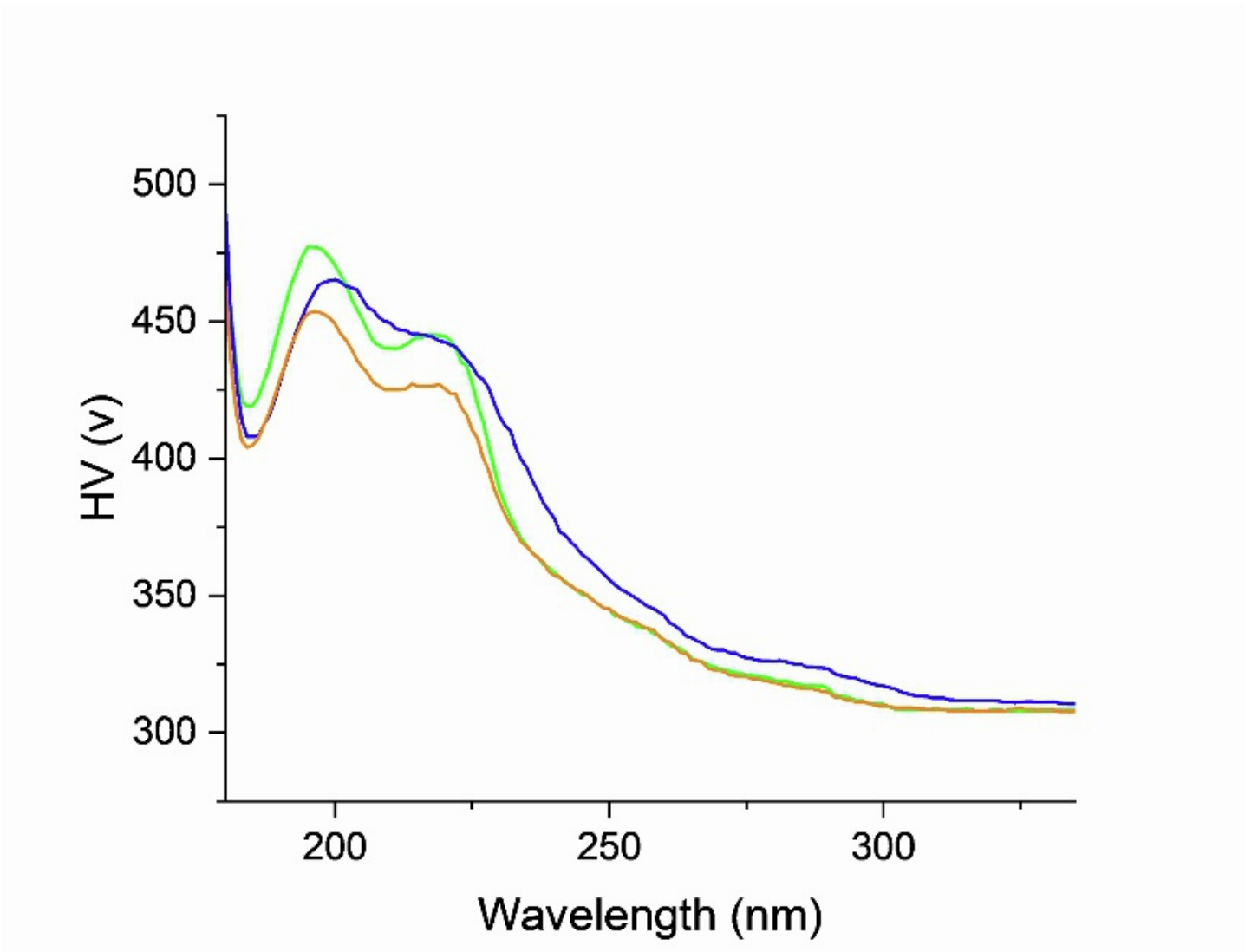

Supplementary Figure 16. Supplementary high voltage (HV) data of CD spectra presented in Figure 2a. HV data of **4a** pH 7 10 mM (orange), **4b** pH 7 10 mM (green) and **4a** + **4b** pH 7 5 mM + 5 mM (blue).

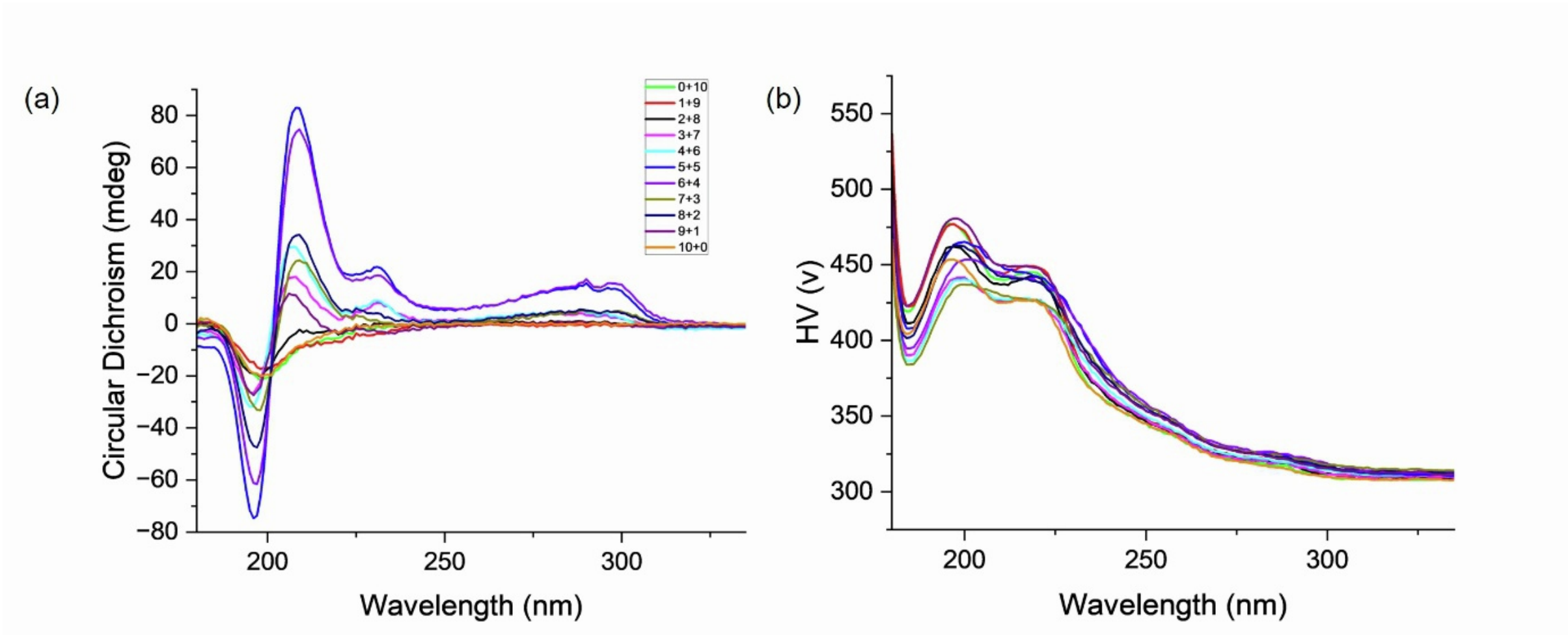

Supplementary Figure 17. Supplementary CD and HV data for the CD Job plot presented in Figure 2b. (a) CD and (b) HV data of **4a** + **4b** pH 7 Job plot solutions at a total concentration of 10 mM. Graph labels correspond to [**4a**] mM + [**4b**] mM.

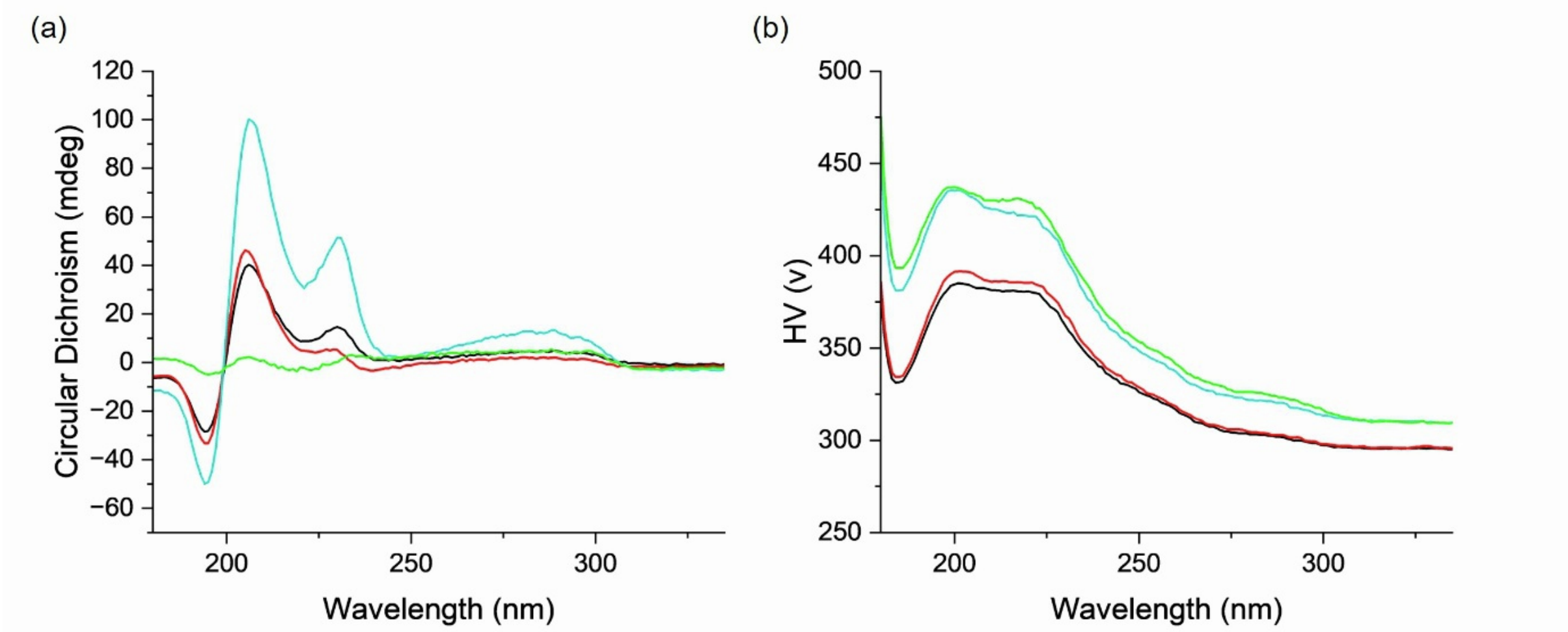

Supplementary Figure 18. CD of altered mixing conditions of 4a + 4b. (a) CD and (b) HV data of **4a** + **4b** pH 7 5 mM + 5 mM 'standard' mixing protocol (black), adjusted up then mix (red) and mix then adjust (turquoise) and after a heat-cool cycle on a rheometer (green), illustrating that each mixing protocol leads to the same peak positions, if different intensities.

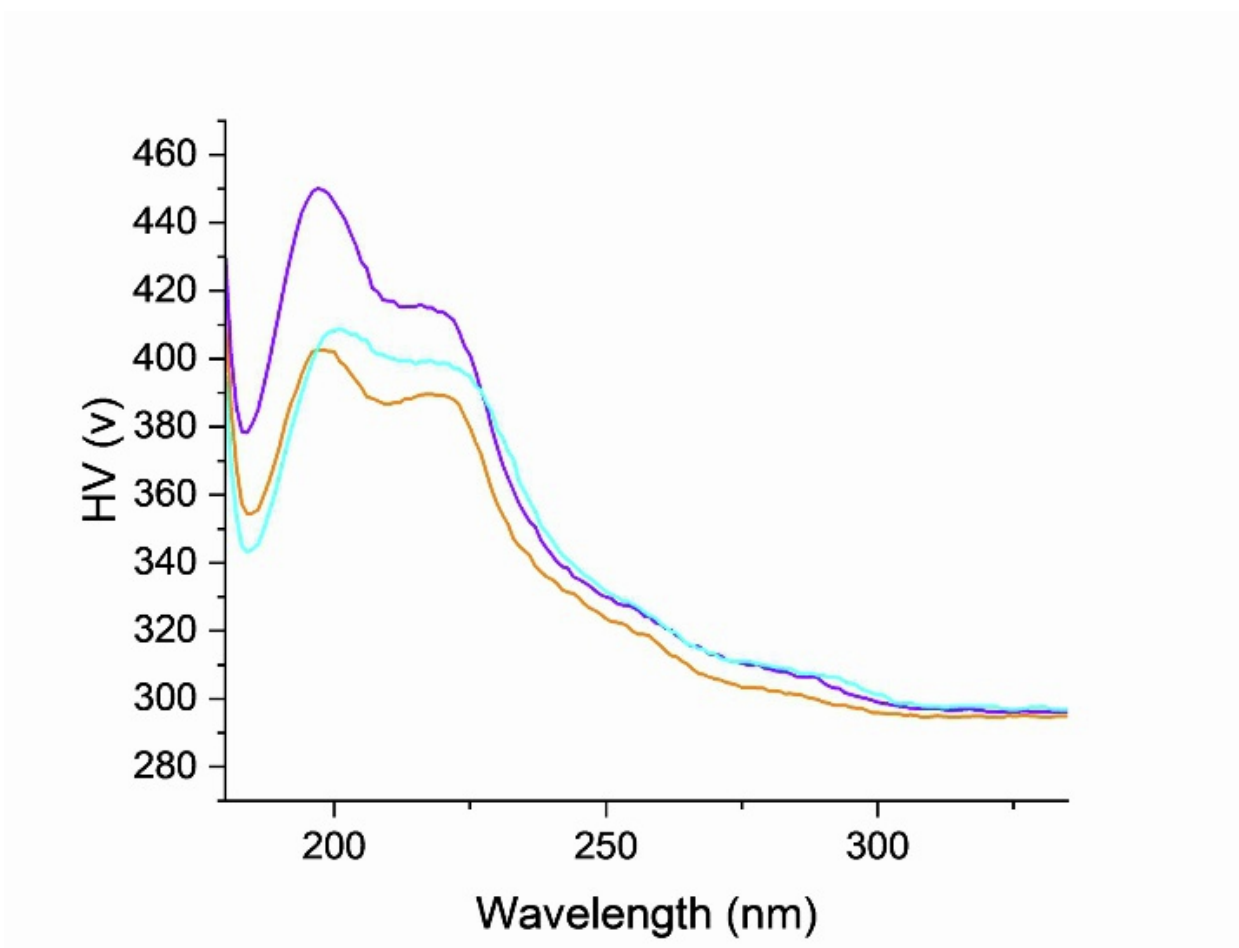

Supplementary Figure 19. Supplementary HV data of the CD presented in Figure 3b. HV data of **4a** pH 7 10 mM (orange), **5b** pH 7 10 mM (purple), and **4a** + **5b** pH 7 5 mM + 5 mM (cyan).

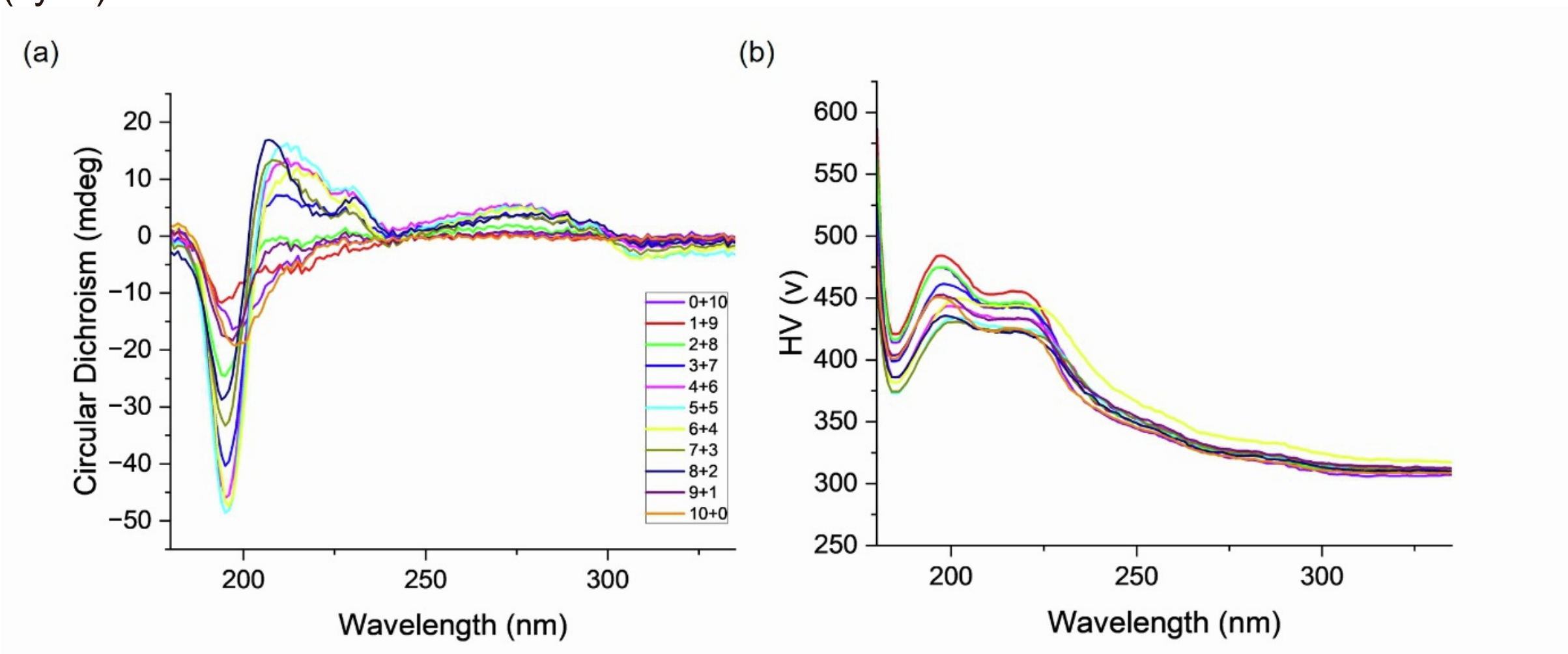

Supplementary Figure 20. Supplementary CD and HV data for the CD Job plot presented in Figure 3c. (a) CD and (b) HV data of **4a** + **5b** pH 7 Job plot solutions at a total concentration of 10 mM. Graph labels correspond to [**4a**] mM + [**5b**] mM.

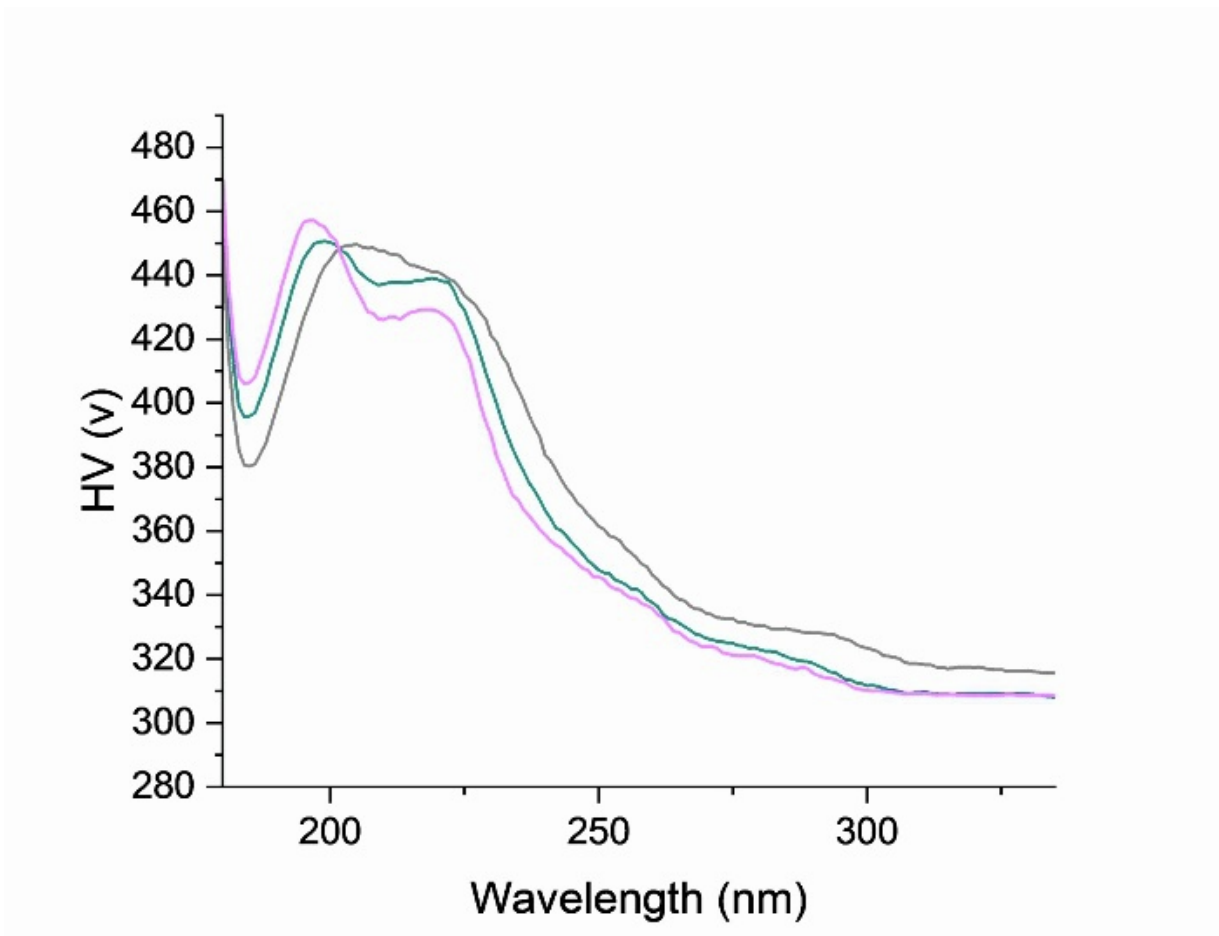

Supplementary Figure 21. Supplementary HV data of the CD presented in Figure 3g. HV data of **6a** pH 7 10 mM (pink), **6b** pH 7 10 mM (olive), and **6a** + **6b** pH 7 5 mM + 5 mM (grey).

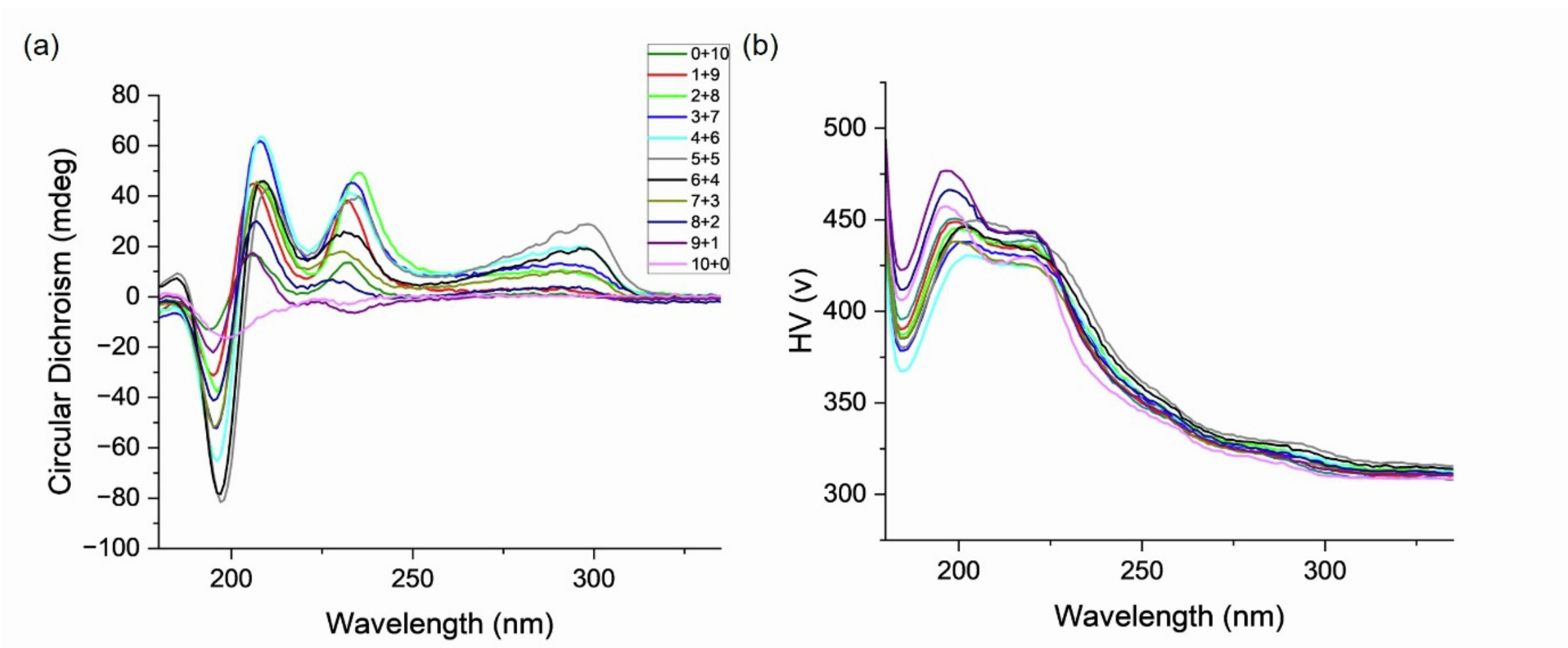

Supplementary Figure 22. Supplementary CD and HV data for the CD Job plot in Figure 3h. (a) CD and (b) HV data of **6a** + **6b** pH 7 Job plot solutions at a total concentration of 10 mM. Graph labels correspond to [**6a**] mM + [**6b**] mM.

Additional experiments

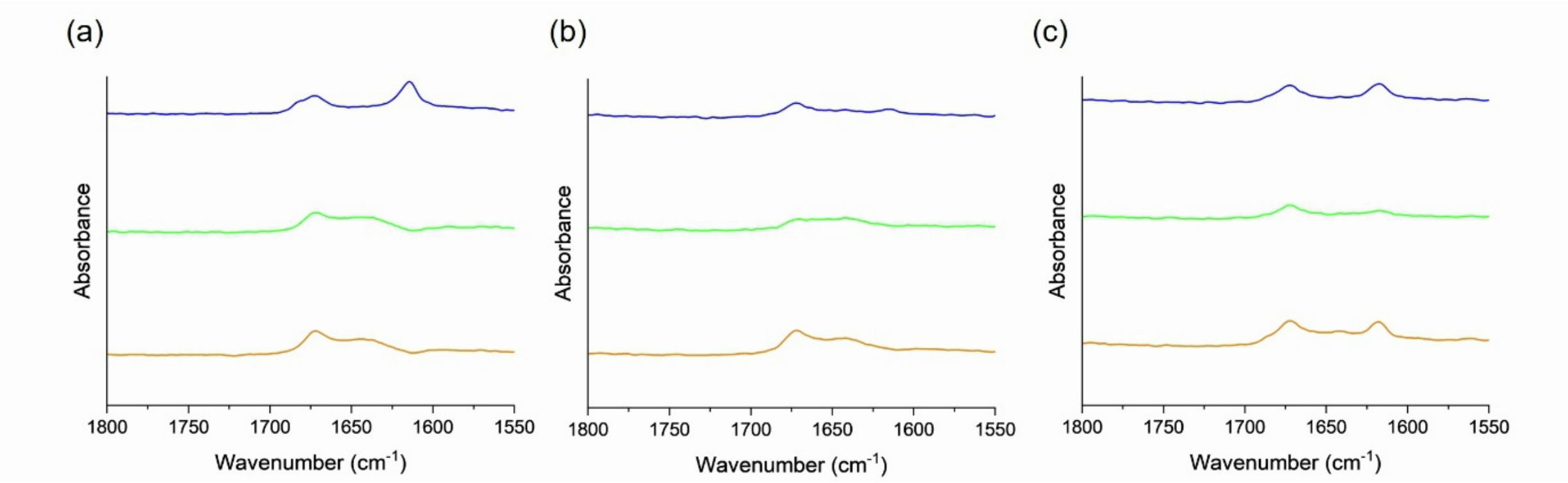

Supplementary Figure 23. FTIR measurements of peptide systems 4, 5 and 6. (a) Fourier-Transform Infra-Red (FTIR) spectroscopy of **4a** pD 7 10 mM (orange), **4b** pD 7 10 mM (green), and **4a** + **4b** pD 7 5 mM + 5 mM (blue). The mixed solution displays the double band characteristic of a β-sheet, while the individual peptides show a small shoulder at 1640 cm^{-1}, characteristic of a random coil. (b) FTIR spectroscopy of **4a** pD 7 10 mM (orange), **5b** pD 7 10 mM (green), and **4a** + **5b** pD 7 5 mM + 5 mM (blue). The signals for **5b** and **4a** + **5b** solutions are much weaker than those observed for the **4a** + **4b** system, which correlated with the weak signals observed by CD. The double peak is still observable in the mixed solution, while individual peptides exhibit the random coil signal. (c) FTIR spectroscopy of **6a** pD 7 10 mM (orange), **6b** pD 7 10 mM (green), and **6a** + **6b** pD 7 5 mM + 5 mM (blue). The double peak signals are present in all three spectra, which is contradictory to the random coil signal observed in the CD of **6b** solutions, however as fibrous structures are observed in cryo-EM images it is not unreasonable that β-like signals are picked up by FTIR.

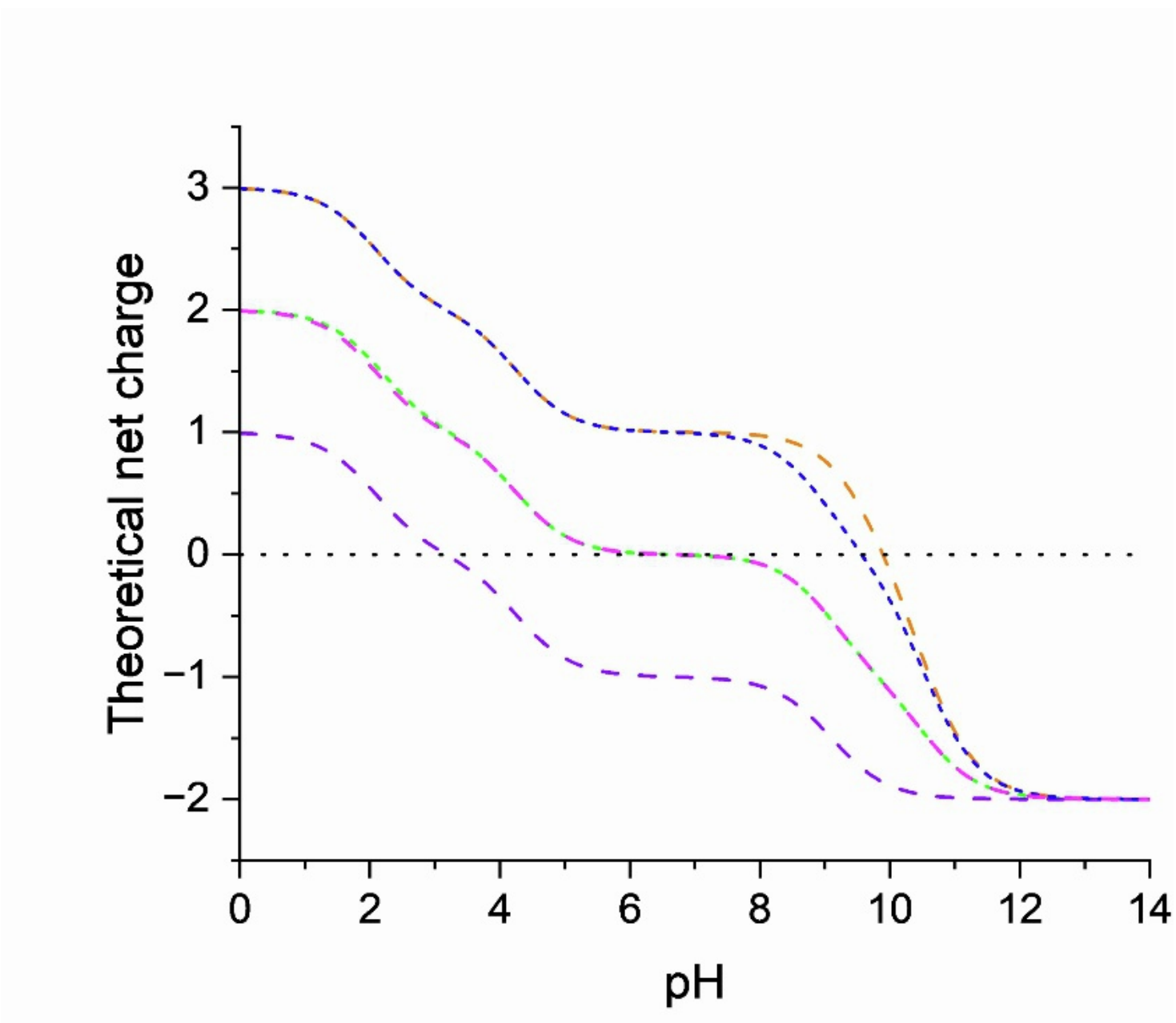

Supplementary Figure 24. Theoretical net charge calculations of peptide systems 4, 5 and 6. Calculated theoretical net charges as a function of pH for **4a** (orange), **4b** (green), **5b** (purple), **6a** (blue) and **6b** (pink). The black dotted line represents net charge = 0. Theoretical net charges were calculated using the equation

$$Z = \sum_i N_i \frac{10^{pKa_i}}{10^{pH} + 10^{pKa_i}} - \sum_j N_j \frac{10^{pH}}{10^{pH} + 10^{pKa_j}} \text{ [S2]}$$

where $N_{i/j}$ are the numbers and $pK_{ai/j}$ the pK_a values of the basic (i) and acidic (j) groups present on the peptide.

Supplementary Tables

Peptide nomenclature

Peptide system	Label	Amino acid sequence
1	**1a**	EVKVEVKVE
	**1b**	KVEVKVEVK
2	**2a**	EVKAEVKAE
	**2b**	KAEVKAEVK
3	**3a**	EVTAKVKAT
	**3b**	TATVEATVK
4	**4a**	EVWAKVKAT
	**4b**	TATVEAWVK
5	**5b**	TATVEAWVT
6	**6a**	KVWAKVEAT
	**6b**	TAKVEAWVT

Supplementary Table 1. Peptide nomenclature scheme. A table relating peptide numbering system to peptide amino acid sequences.

Small angle x-ray scattering

Scattering length density (SLD) was calculated using the NIST neutron activation and scattering calculator (https://www.ncnr.nist.gov/resources/activation/) and the following SLD values were used: deionised H_2O = 9.469 x 10^{-6} $Å^{-2}$, peptide **4a** = 14.182 x 10^{-6} $Å^{-2}$, peptide **4b** = 14.144 x 10^{-6} $Å^{-2}$, peptide **5b** = 14.083 x 10^{-6} $Å^{-2}$, peptide **6a** = 14.182 x 10^{-6} $Å^{-2}$, peptide **6b** = 14.144 x 10^{-6} $Å^{-2}$.

**Model**	**Elliptical cylinder + power law**
**SLD**	14.16
**SLD solvent**	9.469
**Background**	0.39
**A scale**	0.011496<br>(± 3.57E-05)
**A radius (Å)**	55
**A axis ratio**	2.5
**A length (Å)**	5000
**B scale**	0.000151<br>(± 1.18E-06)
**B power**	3.154<br>(± 0.001505)
**Q range ($Å^{-1}$)**	0.00773 – 0.286
**X^2**	3.0803

Supplementary Table 2. Fitting parameters for SAXS data of 4a + 4b pH 7 5 mM + 5 mM shown in Figure 2d.

**Model**	**Elliptical cylinder + power law**
**SLD**	14.14
**SLD solvent**	9.469
**Background**	0.012206

**A scale**	0.000394 (± 6.89E-06)
**A radius (Å)**	110
**A axis ratio**	2
**A length (Å)**	5000
**B scale**	1.92E-06 (± 1.25E-07)
**B power**	3.2093 (± 0.013945)
**Q range ($Å^{-1}$)**	0.00773 – 0.286
**X^2**	1.9146

Supplementary Table 3. Fitting parameters for SAXS data of 4a + 5b pH 7 5 mM + 5 mM shown in Figure 3e.

**Model**	**Elliptical cylinder + power law**
**SLD**	14.14
**SLD solvent**	9.469
**Background**	0.009746
**A scale**	0.000199 (± 1.44E-05)
**A radius (Å)**	25
**A axis ratio**	4
**A length (Å)**	5000
**B scale**	2.28E-05 (± 1.96E-06)

**B power**	2.6548 (± 0.015828)
**Q range (Å^{-1})**	0.00477 – 0.209
**χ^2**	1.2723

Supplementary Table 4. Fitting parameters for SAXS data of 6b pH 7 10 mM shown in Figure 3j.

**Model**	**Elliptical cylinder + power law**
**SLD**	14.16
**SLD solvent**	9.469
**Background**	0.016883
**A scale**	0.00018 (± 1.25E-05)
**A radius (Å)**	55
**A axis ratio**	2.5
**A length (Å)**	5000
**B scale**	1.50E-06 (± 1.41E-07)
**B power**	3.4688 (± 0.01779)
**Q range (Å^{-1})**	0.00494 – 0.235
**χ^2**	1.6364

Supplementary Table 5. Fitting parameters for SAXS data of 6a + 6b pH 7 5 mM + 5 mM shown in Figure 3j.

Supplementary References

[S1] Jumper, J. *et al.* Highly accurate protein structure prediction with AlphaFold. *Nature* **596**, 583–589 (2021).

[S2] Roberts, D., Rochas, C., Saiani, A. & Miller, A. F. Effect of Peptide and Guest Charge on the Structural, Mechanical and Release Properties of β-Sheet Forming Peptides. *Langmuir* **28**, 16196–16206 (2012).
